# Huanglong Antitussive Granule Relieves Acute Asthma Through Regulating Pulmonary Lipid Homeostasis

**DOI:** 10.3389/fphar.2021.656756

**Published:** 2021-04-23

**Authors:** Hua Yan, Guiying Qian, Rui Yang, Zichen Luo, Xianzheng Wang, Tong Xie, Xia Zhao, Jinjun Shan

**Affiliations:** ^1^ Jiangsu Key Laboratory of Pediatric Respiratory Disease, Institute of Pediatrics, Affiliated Hospital of Nanjing University of Chinese Medicine, Nanjing, China; ^2^ Changshu Hospital Affiliated to Nanjing University of Chinese Medicine, Changshu, China; ^3^ Jiangsu Engineering Research Center for Efficient Delivery System of TCM, School of Pharmacy, Nanjing University of Chinese Medicine, Nanjing, China

**Keywords:** acute asthma, treatment, huanglong antitussive granule, lipidomics, pulmonary lipids

## Abstract

**Background:** Asthma is a respiratory disease with chronic airway inflammatory, and individuals with asthma exacerbations is one of the most frequent causes of hospitalization. Huanglong antitussive granule (HL Granule), a Chinese proprietary herbal medicine, has been proved to be effective in the clinical treatment of pulmonary disease. This study is devoted to the pharmacodynamics of HL Granule in acute asthma and the possible mechanism from the perspective of lipidomics.

**Methods:** Mice were divided into four groups, control group, acute asthma model group, HL Granule treatment and montelukast sodium treatment group. Acute asthma was induced by ovalbumin (OVA). Histopathology, pulmonary function and enzyme linked immunosorbent assay (ELISA) were used to validated model and effect of HL Granule. Lipids were detected by ultra-high-performance liquid chromatography coupled to hybrid Quadrupole-Exactive Orbitrap mass spectrometry (UHPLC-Q-Exactive Orbitrap MS) and identified by MS-DAIL and built-in Lipidblast database. Differentially expressed lipids recalled in HL Granule treatment group were extracted for heatmap, enrichment analysis and correlation analysis.

**Results:** HL Granule was effective in decreasing airway hyperresponsiveness (AHR), airway inflammatory and the levels of IL-4 and IL-5. A total of 304 and 167 lipids were identified in positive and negative ion mode, respectively. Among these, 104 and 73 lipids were reserved in HL Granule group (*FDR* < 0.05), including acylcarnitine (ACar), fatty acid (FA), lysophosphatidylcholine (LPC), phosphatidylcholine (PC), lysophosphatidylethanolamine (LPE), phosphatidylethanolamine (PE), phosphatidylglycerol (PG), phosphatidylinositol (PI), phosphatidylserine (PS), diglyceride (DG), triglyceride (TG), sphingomyelin (SM) and ceramide (Cer). Furthermore, 118 and 273 correlations among 47 and 96 lipids in the positive and negative were observed, with ether-linked phosphatidylethanolamine (PEe) and phosphatidylcholine (PCe) (*FDR* < 0.001, Spearman correlation coefficient *r* ^2^ > 0.75).

**Conclusion:** HL Granule might improve pulmonary lipid homeostasis and could be used as an alternative or supplementary therapy in clinical for the treatment of asthma.

## Introduction

Asthma is a common chronic respiratory disease, characterized by respiratory symptoms and airflow limitation that vary over time, with different degrees of chronic airway inflammation and remodeling (Papi et al., 2018). In patients with asthma, acute exacerbation is defined as the changes of previous state, including symptoms, such as wheezing, shortness of breath, chest tightness and cough that recurrence or aggravation with decreasing respiratory function (Kostakou et al., 2019). Environmental determinants, such as allergen, polluted air, smoking, respiratory tract infection, etc, are known to induce asthma (Khreis et al., 2017; Neophytou et al., 2018; Mikhail and Grayson, 2019). Among them, infection is the main cause of acute asthma in children of any age, followed by allergy in school-age children (Dondi et al., 2017). Epidemiological studies show that the global prevalence of asthma is increasing (Lundbäck et al., 2016). The prevalence continues in low-and middle-income countries with lower incidence before, such as China, while may have reached a stable period in high-income countries with high incidence, such as Europe (Lundbäck et al., 2016; Huang et al., 2019; Rodriguez et al., 2019). Acute asthma is mainly a disease occurring in early childhood and the most common cause of hospitalization in children. There were 640,000 children visiting the pediatric emergency department every year, causing serious social and economic burden (Pardue Jones et al., 2016; Saglani et al., 2019). Moreover, acute asthma not only affects the quality of life in children, but also may develop into respiratory failure, even life-threatening if seriously. In Europe, about 15,000 people die a year due to asthma (Fergeson et al., 2017).

Currently, the interventions available for acute asthma remain relatively limited. The commonly used drugs for asthma are β2-adrenergic agonists, corticosteroids (ICSs) and leukotriene modifiers, usually montelukast sodium (Ramsahai et al., 2019). Montelukast sodium, a highly selective leukotriene receptor antagonist, has significantly contributed to asthma control, e.g., reducing asthma severity, especially early wheezing and disease control, over the past 20 years, and was demonstrated decreased peripheral blood eosinophil and induced inhibition of both early and late phase bronchoconstriction in asthma patients. In clinical practice, many doctors prefer montelukast sodium over ICSs (Valentovic, 2007; Lee and Kim, 2020). However, the United States Food and Drug Administration (FDA) recently reminded a black box warning of montelukast sodium, which is severe neuropsychiatric adverse reactions and has been added to the latest Global Initiative of Asthma (GINA) (Glockler-Lauf et al., 2019; Global Strategy for Asthma Management and Prevention Global Initiative for Asthma (GINA), 2020). At present, traditional Chinese medicine (TCM), whether used alone or in combination with conventional asthma medication, has been widely applied to asthma treatment in China. Furthermore, TCM may propose possible explanations to explain asthma heterogeneity. TCM emphasizes the holistic concept and dialectical treatment that the human body is an organic whole, and a disease may have different treatments with different syndrome differentiations. In present study, HL Granule, a Chinese proprietary herbal medicine, has been widely used in various respiratory diseases, such as asthmatic bronchitis, cough variant asthma and other diseases with cough and wheeze (Naiming, 2004; Wenjin, 2018; Yin, 2019). According to the theory of TCM, the reason that asthma recurrent episodes is the phlegm *in vivo* all the time, which induces asthma on the premise of deficiency of healthy qi. HL Granule has the effect of tonifying kidney Qi, cleaning lung, relieving cough, calming panting, as well as ameliorating expectoration of phlegm., which is composed with *Astragalus atropilosulus* (Huangqi), *Epimedium brevicornu* (Yinyanghuo), *Platycodon grandiflorum* (Jiegeng), *Pheretima* (Dilong), *Belamcanda chinensis* (Shegan), *Houttuynia cordata* (Yuxingcao), *Ephedra sinica* (Mahuang), *Crataegus pinnatifida* (Shanzha) and *Lepidium apetalum* (Tinglizi) (Supplementary Table S1), However, no study has explored the efficacy and mechanism of HL Granule in the treatment of asthma. Therefore, in the study, we focused on the pharmacodynamics and possible mechanism of HL Granule while treating acute asthma, and explored the possibility of HL Granule as a supplementary or alternative therapy for montelukast sodium.

Lipidomics is a branch of metabolomics and the separate discipline that studies lipids systematically. Lipids are one of the most important components of living organisms, which can be divided into eight categories, including fatty acids, glycerides, phospholipids, sphingolipids, glycolipids, polyketide, sterols and isoamylenol ester (Fahy et al., 2011; Zhang et al., 2018). Changes in various biological processes will cause changes in lipids. Meanwhile, lipid changes are involved in various biological processes, including energy metabolism, cell membrane-related functions, signal molecules, inflammatory reactions, biomacromolecule-recognition signals, etc. (Barrera et al., 2013). Most of the intracellular life activities occur at the metabolic level, which reflect the environment of cells, and the fluctuation of metabolites represent the complete pathophysiological characteristics including the interaction between heredity and environment (Kelly et al., 2017), while asthma is a complex disease that involves the interplay of genes and environmental factors. Studies have shown that lipid metabolism of asthmatic patients in bronchoalveolar lavage fluid (BALF) is disturbed (Kang et al., 2014). Lipids with significant changes, on the one hand, may be used as biomarkers (Ackerman et al., 2016; Trinh et al., 2016); on the other hand, play an important role in innate immunity and inflammation (MacEyka and Spiegel, 2014; O’Donnell et al., 2018). Therefore, the pharmacodynamic mechanism of HL Granule in an acute model of asthma was studied on the basis of lipidomics.

In present study, firstly, we detected pulmonary function, histopathology and inflammatory factors to evaluate the model and the curative effect of HL Granule. Afterward, on the basis of established stable and effective lipid analysis method (Yang et al., 2019), we used liquid chromatography-mass spectrometry (LC-MS) technology to research the changes of lipids in the lung tissue. Lastly, we constructed a lipid map of acute asthma and analyzed the regulatory effect of HL Granule on disordered lipids to explore the possible pharmacodynamic mechanism of HL Granule in the treatment of acute asthma.

## Methods and Materials

### Chemicals and Regents

HL Granule (Lot No, 190107121) was purchased from Shanxi Dongke Pharmaceutical (Shanxi, China). Montelukast Sodium Oral Granule (Lot No, 829966) was purchased from Mercksharp and DohmeCorp (Inc. Kenilworth, NJ, United States). Standards of astragaloside IV (Lot No, 140321), icariin (Lot No, 18012906), irisflorentin (Lot No, 20041001), citric acid (Lot No, 19061002), quercetin 3-O-β-D-glucose-7-O-β-D-gentiobioside (Lot No, 19112806), deapio-platycodin D (Lot No, 19070307), platycodin D2 (Lot No, 19070306), deapio-platycodin D2 (Lot No, 19070308) and polygalacin D (Lot No, 170623) were from Chengdu Pufei De Biotech (Chengdu, China). Standards of astragalosid I (Lot No, 120525), astragaloside II (Lot No, 120528), calycosin (Lot No, 120615), calycosin-7-O-beta-D-glucoside (Lot No, 120819) and platycodin D (Lot No, 120826) were from Chengdu Herbpurify (Chengdu, China). Standards of ephedrine hydrochloride (Lot No, 171241-200506) and pseudoephedrine hydrochloride (Lot No, 171237-200806) were from National Institutes for Food and Drug control (Beijing, China). Standards of 3-O-β-D-glucopyranosyl platycodigenin (Lot No, P27A9S60106), platycodin D3 (Lot No, P28M8F32837) and platycoside E (Lot No, P06M10S81977) were from Shanghai Yuanye Biotech (Shanghai, China). The standard of deapio-platycoside E/platycoside g1 (Lot No, FY37239B0234) was purchased from Nantong Feiyu Biotech (Nantong, China). Ovalbumin (OVA) was obtained from Sigma-Aldrich (St. Louis, MO, United States).

High performance liquid chromatography (HPLC) grade acetonitrile, methanol, formic acid, ammonium formate and ammonium acetate were purchased from Merck (LiChrosolv, Merck, Darmstadt, Germany). MS grade isopropanol was from ROE Scientific (United States). Standards of lysoPE (17:1) (Lot No, LM171LPE-11), SM (17:0) (Lot No, 170SM-13), TG (17:0/17:1/17:0 D5) (Lot No, 170/171/170TG(DG)-10) and PE (17:0/17:0) (Lot No, LM170PE-19) were obtained from Avanti Polar Lipids (AL, United States).

### Components’ Detection of HL Granule

HL Granule was dissolved in double distilled water, and ultrasound extracted for 30 min by methanol. The extraction was centrifuged by 12,000 rpm/min at 4°C for 10 min, and supernatant was injected into the instrument for components’ detection. Chromatographic separation was performed on Waters ACQUITY UPLC H-CLASS system (Waters, Milford, MA, United States) with a AQUITY UPLC HSS T3 column (2.1 × 100 mm, 1.8 *μ*m, United States). The column compartment was maintained at 35°C. The mobile phase was consisted of solvent A (0.1% formic acid in water) and solvent B (0.1% formic acid in acetonitrile). Gradient conditions were as follows: 0 min, 10% B, 0–23 min, 10%-90% B, 23–24 min, 90% B, 24–26 min, 90%-10% B, 26–30 min, 10% B, at a flow rate of 0.2 ml/min.

Mass spectrometry data was acquired by LTQ ORBITRAP XL mass spectrometer system (Thermo Scientific, United States) both in the positive and negative ionization mode. The temperature of the heated capillary was 270°C and that of the ESI probe was 300°C. The flows of sheath gas and auxiliary gas were set to 40 and 10 units, respectively. The scan range of the Orbitrap analyzer was *m/z* 500–1800 and MS^n^ data were acquired by LTQ in data dependent acquisition mode. Both of the positive and negative adopted the same MS parameters.

### Experimental Animals

Female C57BL/6J mice (6 weeks old) were purchased from Nanjing Qinglongshan animal farm (Certificate number 201909516, Jiangsu, China). All the animal experiments were approved by the Animal Care and Use Committee of Nanjing University of Chinese medicine (Permit number: 201903A018). Before experiments, mice were housed in a facility free of specific pathogens and had one-week adjustment period.

**FIGURE 1:**
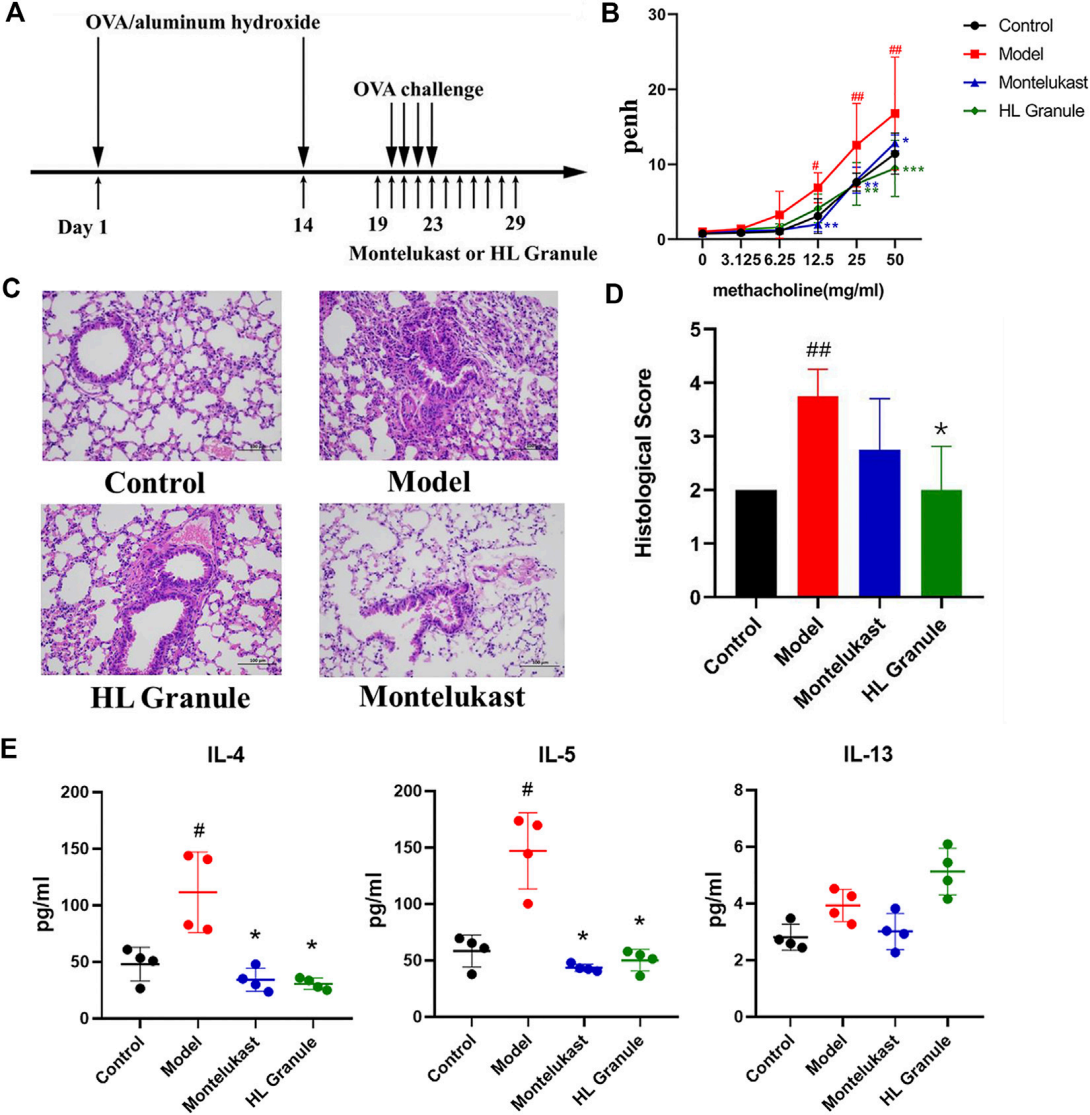
The protective effect of HL Granule on acute asthma in mice. **(A)** The flowchart of induced and treatment in the model of acute asthma. The model of acute asthma was established and sensitized by intraperitoneal injection of 0.2 ml OVA, followed by atomization of 3% OVA. **(B)** Pehn in each group. The data was collected in response to gradient concentration (0, 3.125, 6.25, 12.5, 25, and 50 mg/ml) of inhaled MCh. **(C, D)** Representative HE staining pictures (x200, C) and histological scores, *n* = 4. **(E)** The expressions of IL-4, IL-5 and IL-13 in BALF by Elisa. Results are presented as mean ± SD, statistics in **(B)** was using two-way ANOVA, *n* = 3; statistics in D and E were using Kruskal-Wallis test, *n* = 4; #*p* < 0.05, ##*p* < 0.01 compared to control group; *p < 0.05, compared to model group.

Mice were divided into four groups, ten mice per group: control group, acute asthma model group, HL Granule treatment group and montelukast sodium treatment group. Acute asthma model was induced by ovalbumin (OVA). In detail, on the first and 14th day, mice were sensitized by intraperitoneal (IP) injection with 0.2 ml sensitizing solutions, containing 20 µg OVA and 1 mg aluminum hydroxide. From day 20, mice inhaled 3% (wt/vol) atomized OVA for 30 min by using an ultrasonic nebulizer (Yuwell, Nanjing, China) for four consecutive days. On the 19th day, model mice were divided into three groups according to different treatments, i.e., model group, HL Granule group and montelukast sodium group, and individually oral gavaged with double distilled water (0.1 ml/10 g) (model group), HL Granule (9.1 g/kg/d) (HL Granule group), and montelukast sodium (1.52 mg/kg/d) (montelukast sodium group) at 24 h intervals for seven consecutive days. Throughout the experiment, control group was given IP injections and inhalation of saline following the same procedure as acute asthma model, and was administered by gavage with double distilled water (0.1 ml/kg) from day 19–25. The procedure has been summarized in Figure 1A.

### Histopathology and Biochemical Analysis

*Histopathology.* Lung tissue sections were stained with hematoxylin and eosin (HE) to investigate the pathological changes. Histological scores were graded from 0 (normal) to 4 (severe) based on Inflammatory cell infiltrated in peribronchiolar and alveolar wall, degeneration, necrosis or exfoliation of bronchial epithelium, the integrity of alveolar wall, mucus secretion and goblet cell proliferation.

*Pulmonary function analysis.* Pulmonary function was evaluated by Whole Body Plethysmography (Emka Technologies, France) after the last administration. The airway was stimulated with increasing concentrations of erosolized methacholine (MCh) (0, 3.125, 6.25, 12.5, 25, 50 mg/ml). Then, enhanced pause (Penh) was determined to evaluate the bronchoconstriction function.

*Enzyme linked immunosorbent assay (ELISA).* ELISA kits were used to detect the expression of Interleukin-4 (IL-4), Interleukin-5 (IL-5) (Biolegend, San Diego, CA) and Interleukin-13 (IL-13) (Yi Fei Xue Biotechnology, China) in BALF.

### Statistical Analysis

Data are expressed as mean ± standard deviation (SD) in scatter plots and column bar graphs. Box-whisker Plot shows median, lower, and upper quartiles, as well as minimum and maximum values. GraphPad Prism 8.0.2 (GraphPad Software, United States) was used to analyze data by Mann-Whitney non parametric test for two unmatched groups and one-way ANOVA or two-way ANOVA for multiple comparisons. Enrichment *p*-values are given by the Kolmogorov-Smirnov-test. *p* < 0.05 is considered significant.

### Untargeted Lipidomics Analysis With UHPLC-Q-Exactive Orbitrap MS

*Lipid Extraction for Detection.* A total of 20 mg lung tissue was used to extract lipids by the MTBE/MeOH/H2O system. First, tissue homogenate was extracted by 225 μl ice methanol solution containing internal standards (LPE (17:1), SM (17:0), TG (17:0/17:1/17:0) and PE (17:0/17:0), 5 μg/ml). After vortexing for 10 s, 750 μl MTBE was added, and the mixture was vortex for 10 min. Subsequently, 188 μl of deionized water was added and the mixture was vortexed for 20 s. After centrifuging 2 min by 14,000 rpm at 4 C, the upper phase was dried in a vacuum concentrator. Finally, the upper phase lipid was reconstituted in the mixed solvent of methanol: toluene (9:1, v/v) for lipidomic analysis.

*Quality Control Samples.* The preparation was carried out by mixing equal aliquots of 5 μl from each sample, and their pretreatment was carried out in the same manner as the samples. Five QCs were injected before samples, while one QC injection was inserted regularly after every 10 samples.

*Chromatographic separation conditions.* Untargeted lipidomics analysis of lung tissue was performed on an Ultra-high-performance liquid chromatography (Thermo Fisher Scientific, United States) coupled to hybrid Quadrupole-Exactive Orbitrap mass spectrometry (Thermo Fisher Scientific, United States). A reversed phase Waters Acquity UPLC CSH C18 (100 mm × 2.1 mm, 1.7 μm) maintained at 60°C was used for the chromatographic separation of lipids. The flow rate was set at 0.3 ml/min. Mobile phase A were ACN/H2O (6:4, v/v), and B IPA/ACN (9:1, v/v), both containing 10 mM ammonium formate and 0.1% formic acid for positive ionization mode. For the negative ionization mode, 10 mM ammonium acetate was used as buffer system. The elution gradient started with 15% B for 4 min, and then increased from 15 to 48% B in 1 min, from 48 to 82% B in 17 min, and from 82 to 99% B in 1 min, and maintained for 1 min, back to 15% B at 24.2 min and maintained for 5.8 min to equilibrate column.

*Mass Spectrometer Conditions.* The mass spectrometer was operated with the following parameters: spray voltage 3.5 kV (positive) and 3.0 kV (negative). For both ionization modes, the sheath gas and aux gas were separately maintained at 35 and 15 arbitrary units, while the capillary temperature and the heater temperature were 325 and 300°C, respectively. The MS/MS data was acquired by data dependent method and top 10 abundant ions were used for fragmentation. The normalized collision energy (NCE) was set 25, 35, and 45 eV, respectively.

*Data processing of lipidomics.* Data deconvolution, lipid identification and alignment were analyzed by MS-DIAL 3.3 (MS-DIAL software, Japan). The output data matrix was exported for further statistical analysis. MetaboAnalyst 4.0 (metaboanalyst.ca/faces/ModuleView.xhtml) was used for multivariate analysis. Principal component analysis and heatmap were performed for cluster and visulation. Furthermore, lipid metabolites enrichment analysis was based on ChemRICH (http://chemrich.fiehnlab.ucdavis.edu/) (Barupal and Fiehn, 2017). The differentially expressed lipids were screened using the Kruskal-Wallis test and Mann-Whitney test by R 3.6.3. Correlation analysis was conducted using Spearman correlation and visualized with Cytoscape 3.8.2 (Cytoscape software, United States).

## Results

### Quality Control of HL Granule by UHPLC-ESI/LTQ-Orbitrap-MS

**FIGURE 2:**
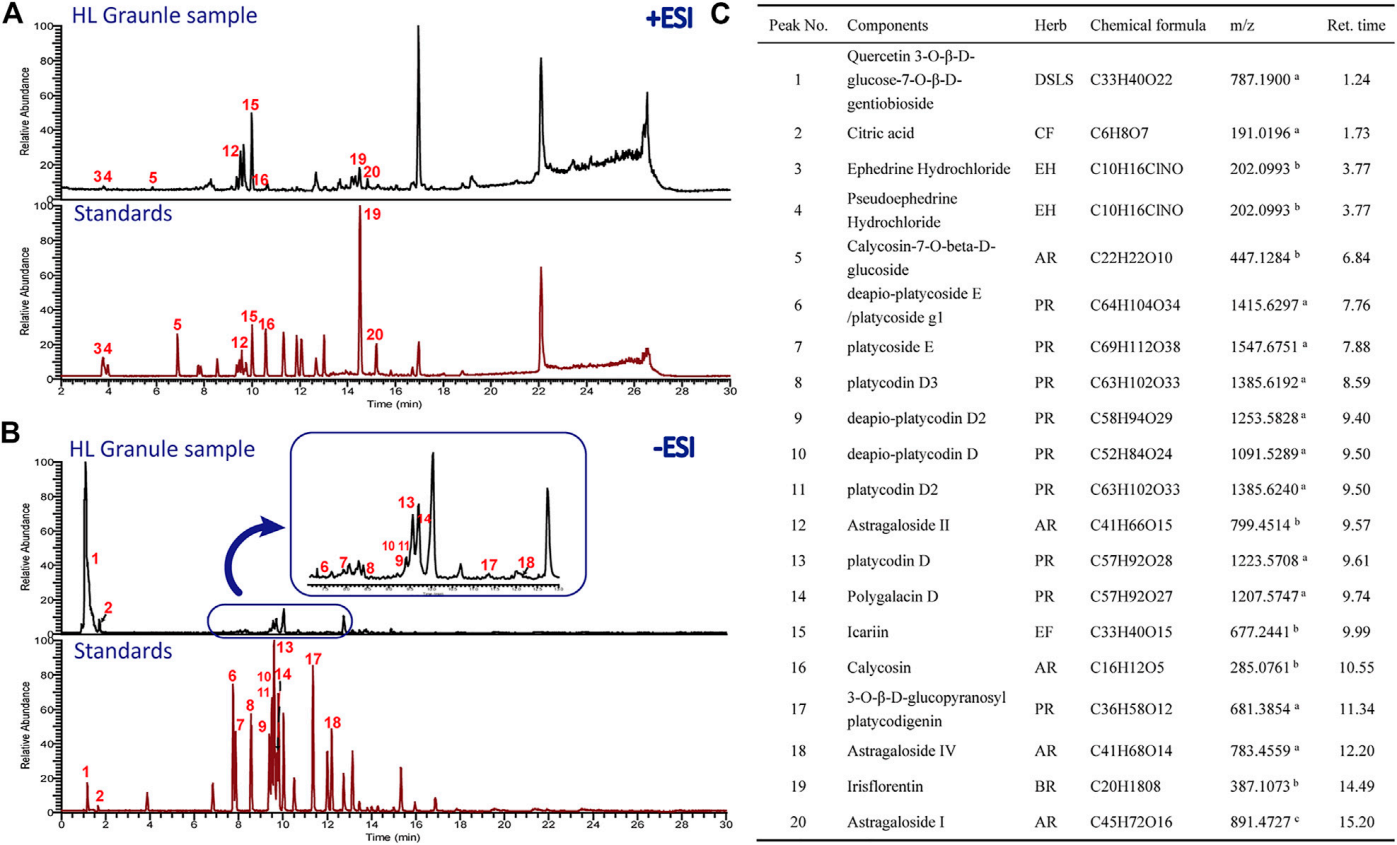
Chromatogram of HPLC analysis of HL Granule extract. **(A, B)** TICs detected in positive **(A)** and negative ion mode **(B)**. **(C)** Major chemical and effective components identified from HL Granule extract. In A and B, the upper of each picture is the chromatograms of the HL Granule sample, while the lower is the mixed standard reference compounds. Numbers in red corresponds to the chemical components in **(C)**. AR: Astragali Radix (*Astragalus atropilosulus*); BR: Belamcandae Rhizoma (*Belamcanda chinensis*); CF: Crataegi Fructus (*Crataegus pinnatifida*.); DSLS: Descurainiae Semen Lepidii Semen (*Lepidium apetalum*); EF: Epimedii Folium (*Epimedium brevicornu*); EH: Ephedrae Herba (*Ephedra sinica*); PR: Platycodonis Radix (*Platycodon grandiflorum*). a [M − H]−; b [M + H]+; c [M + Na]+.

The chemical profile of HL Granule was firstly analyzed by UPLC-ESI/LTQ-Orbitrap-MS. Both of the positive and negative ionizations were used. A total of 20 components belonging to seven crude materials were qualitative identified based on the standard compounds (Figure 2). Flavones and saponins in *Astragalus atropilosulus*, saponins in *Platycodon grandiflorum*, and alkaloids in *Ephedra sinica* were considered as the effective components. The typical total ion chromatograms (TICs) are shown in Figure 2. The results laid a foundation for the effectiveness and safety of HL Granule used in this study for asthma treatment.

### Curative Effect of HL Granule in The Treatment of Acute Asthma in Mice

To develop an acute asthma like symptoms, OVA was used for sensitization and challenge (Figure 1A). At the end of the challenge period, airway hyperresponsiveness (AHR) was evaluated by calculating the enhance pause (Penh). The results showed that Penh increased significantly in model group (*p* < 0.05, 12.5 mg/ml methacholine; *p* < 0.01, 25 mg/ml, 50 mg/ml methacholine), and HL Granule could reverse AHR (*p* < 0.01, 25 mg/ml, 50 mg/ml methacholine, Figure 1B). The results suggested that HL Granule inhibited the bronchoconstriction and improved AHR. Then, inflammatory cell infiltration and airway remodeling were observed by histopathological examination. The results demonstrated that the inflammatory cell infiltration around the trachea and alveolar wall were more serious in the model group without airway remodeling, while HL Granule and montelukast sodium could relieve the inflammatory cell infiltration (Figure 1C). The HE staining was scored semi-quantitatively based on inflammatory cell infiltrated, mucus secretion and goblet cell proliferation mainly in airway and alveoli. Histopathological scores confirmed that HL Granule ameliorated OVA induced inflammatory changes and significantly reduced total leukocyte counts (Figure 1D). Moreover, the curative effect of HL Granule was slightly better than montelukast sodium in alleviating lung pathology (Figure 1D).

T helper 2 (Th2) cell-mediated immunity is considered as an important role in the pathogenesis of asthma. Eosinophils and CD4^+^ cells producing interleukin-5 (IL-5) are frequently found in the blood and lung lavage fluid. To observe the Th2 cell mediated immunity, the levels of IL-4, IL-5, and IL-13 in BALF were analyzed in acute asthma model and the curative effect of HL Granule. The results showed that the levels of IL-4 and IL-5 increased significantly in asthmatic mice (*p* < 0.05). Administration of HL Granule significantly reduced IL-4 and IL-5 in BALF by 65.9 and 72.5% compared with those of the OVA groups (*p* < 0.05) (Figure 1E). In summary, the results suggested that HL Granule could be used in acute asthma treatment, and might be considered for replacing montelukast sodium as an adjuvant treatment of asthma. However, there was no significant change to IL-13.

### Lipidomic Profiling of HL Granule Treatment of Acute Asthma

**FIGURE 3:**
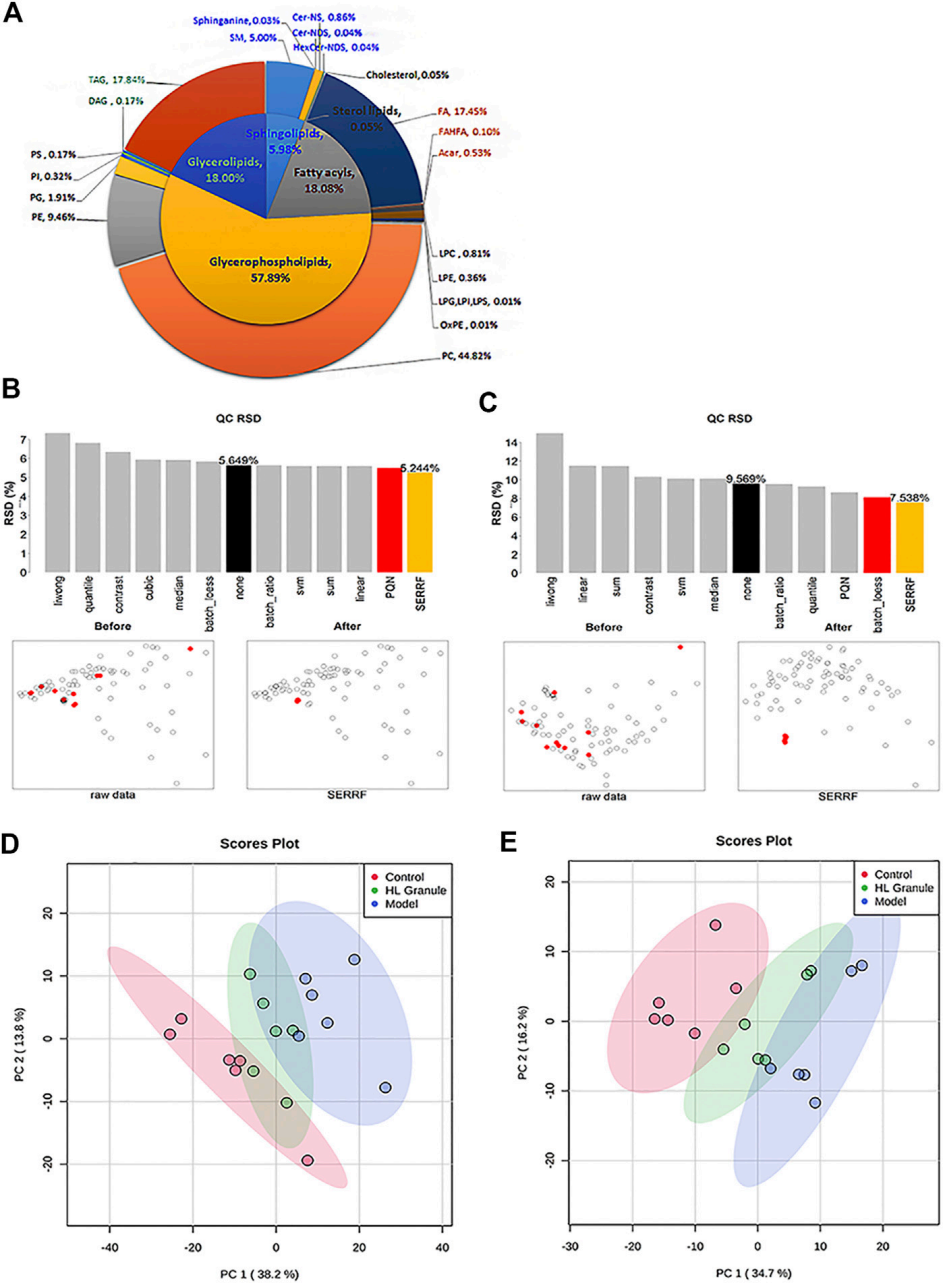
lipids related to HL Granule treatment of acute asthma. **(A)** Double pie chart of lipids detected in lung tissue of acute asthma mice in positive and negative ion mode. **(B, C)** RSD and scatter plots of lipids in samples and QCs after normalization in positive **(B)** and negative ion mode **(C)**. **(D, E)** PCA of lipids in lung of mice in positive (D) and negative ion mode **(E)**, *n* = 6.

Lipids play a central role in lung physiology and pathology. The role of lipids in lung and respiratory disease has attracted more attention in recent years, including cystic fibrosis, asthma and COPD, which are all associated with abnormal metabolism. To examine the lipid changes of OVA induced asthmatic mice, the lipid profile of lung tissue was analyzed. A total of 304 and 167 lipid molecular species sorted into five lipid categories and 22 lipid subclasses were confidently annotated based on the database matching in the positive and negative ion mode (Figure 3A). The most commonly identified lipid species in the developing method belonged to glycerophospholipids, which were dominant with 57.89% in total. Glycerophospholipids were found in the highest amounts in the membranes of all cells. Glycerolipids were account for 18.00%, which are considered as the quantities in fat stores. Additionally, sphingolipids were minor components, and only account for 5.98%. In summary, glycerophospholipids, sphingolipids, fatty acyls and glycerolipids were all important lipids in lung tissue of acute asthma.

Then, pooled QC samples were used to monitor the analytical performance. In order to evaluate and remove the systematic error, different normalization methods were applied after peak picking up and lipid annotation. The results showed the QC samples were clustered together by the random forest (SERRF) method, indicating that a robust data matrix can be obtained by the SERRF method (Figures 3B,C). Then, PCA models were applied to the positive and negative lipidomic data respectively. For the positive lipidomic data, the first two components explained about 50% variables (PC1 = 38.2%, PC2 = 13.8%). For the negative lipidomic data, the first two components explained about 50.9% variables (PC1 = 34.7%, PC2 = 16.2%). The PCA score plots indicated that the component one can distinguish the observations of control and acute asthma obversions (Figures 3D,E). Remarkably, all the observations of HL Granule treatment appeared to cluster from the model group to the control group, suggesting that HL Granule could regulate lipids disorder in lung tissue of OVA induced asthmatic mice. The score plot from negative data showed the same cluster profiles.

### Characterization of Differentially Expressed Lipids

**FIGURE 4:**
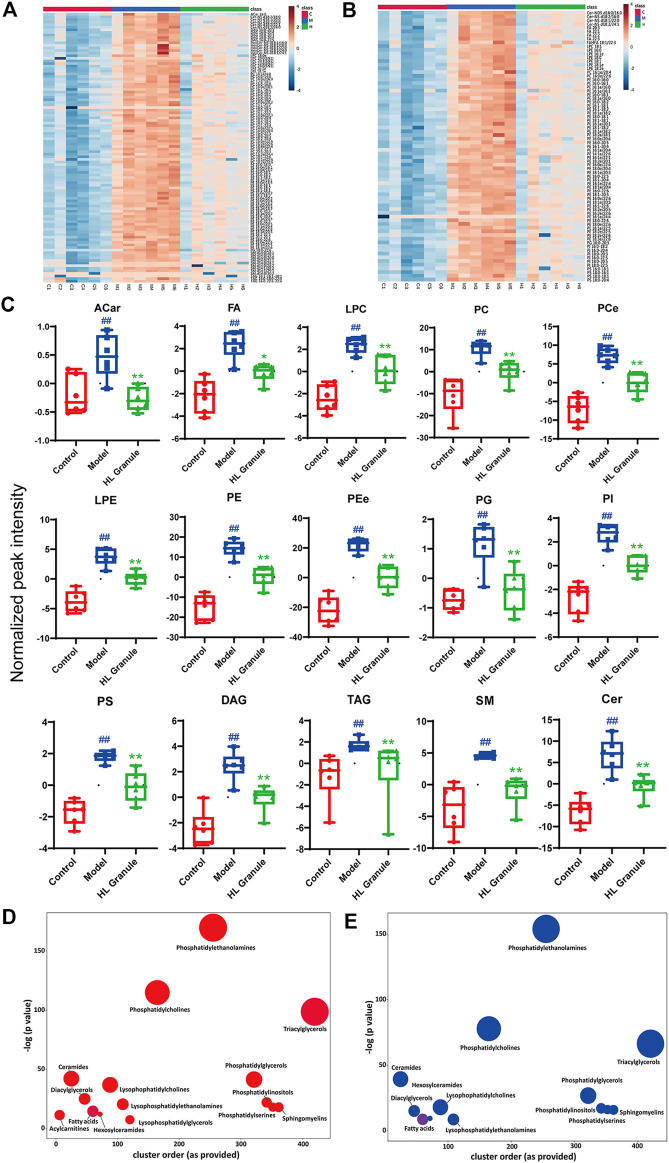
Summary of differentially expressed lipids. **(A, B)** Heatmaps of differentially expressed lipids in positive **(A)** and negative **(B)** ion mode, *n* = 6. Each column represents a sample, each row represents a differential lipid, and each block represents the corresponding intensity value, red to blue of the corresponding lipid in each sample, representing the value from large to small. Differentially expressed lipids were reversed in HL Granule group compared to model group (FDR < 0.05). **(C)** Box-whisker Plot of differentially expressed lipid subclasses, in positive and negative ion mode, *n* = 6 **(D, E)** Enrichment statistics plot of significantly regulated lipids between model and control groups **(D)** as well as HL Granule and model groups **(E)**, *n* = 6. Each node reflects a significantly altered cluster of lipids. Enrichment *p*-values are given by the Kolmogorov-Smirnov-test. Node sizes represent the total number of lipids in each cluster set. The node color scale shows the proportion of increased (red) or decreased (blue) compounds. Purple-color nodes have both increased and decreased lipids. Results in (C) extends from 25th to 75th percentile and the line represents the median, the Mann-Whitney test was used between two groups, *n* = 6; ##*p* < 0.01 compared to control group; **p < 0.01, compared to model group.

To compare the inter groups, the Kruskal Wallis test was used. Correspondingly, to verify the hypothesis about the irrelevance of differences between two groups, the Mann-Whitney test was used. A total of 162 lipids in the positive and 109 lipids in the negative were significantly changed after the OVA induced asthma, including acylcarnitine (ACar), fatty acid (FA), lysophosphatidylcholine (LPC), phosphatidylcholine (PC), lysophosphatidylethanolamine (LPE), phosphatidylethanolamine (PE), phosphatidylglycerol (PG), phosphatidylinositol (PI), Phosphatidylserine (PS), diglyceride (DAG), triglyceride (TAG), sphingomyelin (SM) and ceramide (Cer). The lipids were considered upregulated if fold change (FC) > 2 and FDR < 0.05, and downregulated if FC < 0.5. The results from nonparametric tests showed that the lipid levels of OVA groups were significantly increased compared to control groups. Whereas, treatment of HL Granule significantly reduced the lipid levels. The differentially expressed lipids were illustrated by heatmaps (Figures 4A,B, Supplementary Tables S2, S3). Each square represents the intensity value of a specific lipid in a sample. The transition in color from red to blue represents the intensity value becoming smaller. The darker the red is, the larger the value is, and vice versa. As shown in heatmaps, under the condition of FDR <0.05, a total of 104 lipids upregulated in the model group were reversed, including ACar, LPC, PC, LPE, PE, PG, DG, TG, SM and Cer, in positive ion mode (Figure 4A, Supplementary Table S2). In negative ion mode, a total of 73 lipids were found downregulated (FDR < 0.05), including FA, LPC, PC, LPE, PE, PI, PS, and Cer (Figure 4B, Supplementary Table S3). Box-whisker Plots of 15 major subclasses of differentially expressed lipids were shown, which confirmed the significant altered in lipids among control, model and HL Granule groups in lung tissue (Figure 4C). HL Granule could significantly downregulate the up-regulated lipids in model group by regulating the disorder of ACar, FA, LPC, PC, LPE, PE, PG, PI, PS, DG, TG, SM, Cer, as well as ether-linked phosphatidylethanolamine (PEe) and phosphatidylcholine (PCe) in acute asthma.

Enrichment analysis helps to gain mechanistic insight into metabolite lists. As shown in Figures 4D,E, each node represents a significantly changed lipid group (*p* < 0.05) and the node size reflects the total number of lipids contained in each lipid group. The results showed that phospholipids, sphingolipids, fatty acids and glycolipids changed significantly. Among them, phospholipids, including PE, PC, and TG, contained the higher number of lipids and changed more significantly, suggesting that lipids were important in acute asthma, especially phospholipids. More specifically, compared with the control group, 15 lipid classes in the model group changed significantly (Figure 4D). Apart from fatty acids and triglycerides with partial downregulation, the other lipid classes showed an overall upward trend. After HL Granule treatment, 13 of them were recalled (*p* < 0.05) with overall downregulation and partial upregulation in fatty acids (Figure 4E).

Combined with the results of lipid molecular species and lipid subclasses., we speculated that HL Granule might regulate phospholipids, sphingolipids, fatty acids and glycolipids, especially phospholipids in lung tissue to relieve acute asthma. Therefore, we speculated that HL Granule may improve acute asthma by regulating lipids homeostasis in lung tissue.

### Correlation of the Differentially Expressed Lipids

**FIGURE 5:**
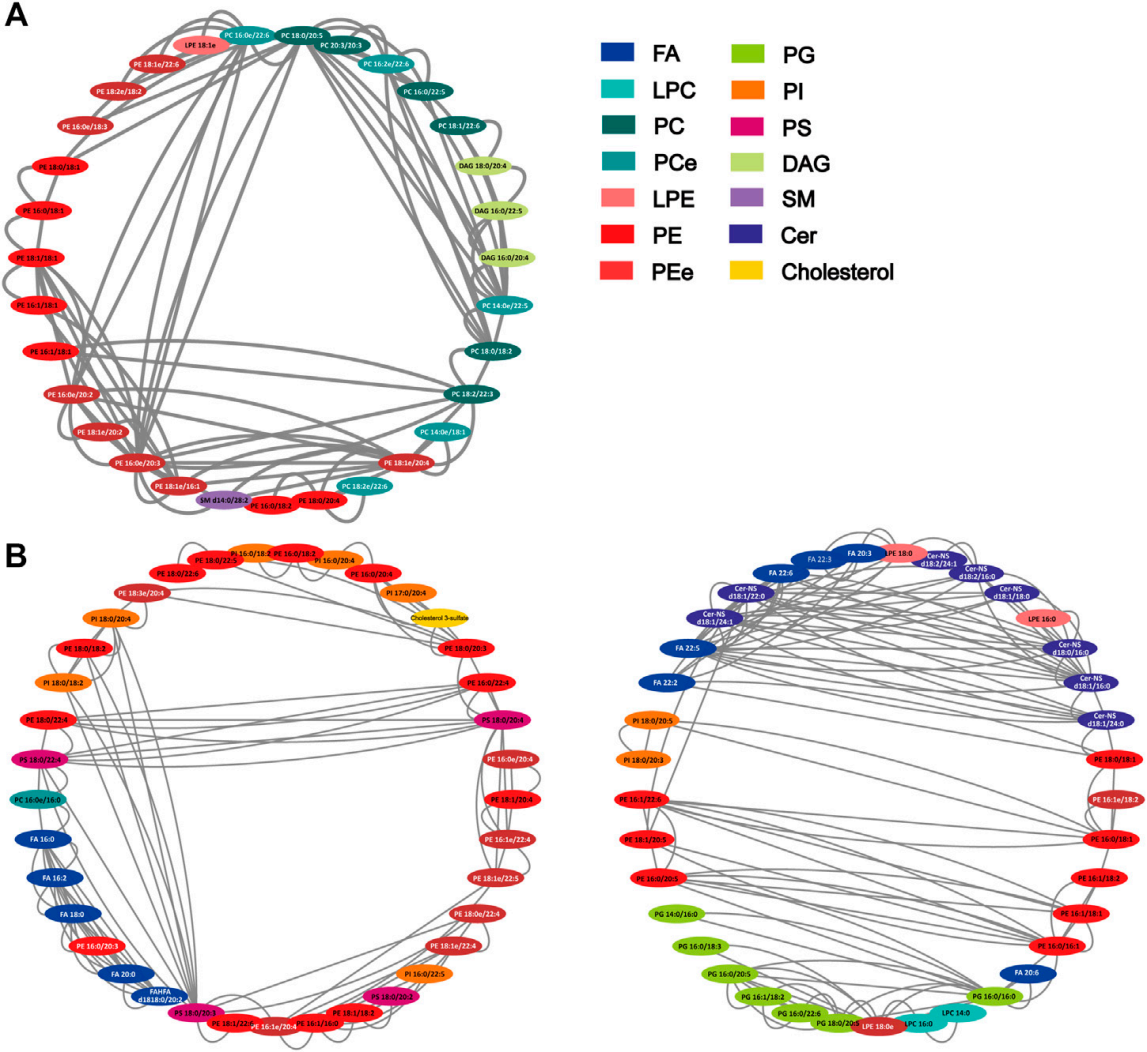
Correlation network of differentially expressed lipids. **(A)** positive ion mode (47 nodes, 118 edges); **(B)** negative ion mode (96 nodes, 273 edges). The network was generated by Cytoscape 3.8.2 with attribute circle layout. Nodes were screened by FDR value <0.001 and Spearman correlation coefficient r^2^ > 0.75. Edges represent the value of r^2^ between two nodes.

Spearman correlation was conducted for all differentially expressed lipids. On the basis of *FDR* < 0.001 and Spearman correlation coefficient *r* ^2^ > 0.75, 118 and 273 notable correlations among 47 and 96 lipids in the positive and negative were observed, with PEe and PCe. As shown in Figure 5A, of the 118 edges, PE (16:0e/20:3), PC (18:0/20:5), PE (18:1e/20:4), PC (14:0e/22:5) and PC (16:0e/22:6) showed significant correlation (edges ≥ 12). PC and PE are the most abundant phospholipids in all mammalian cell membranes, which can contain ether- bonds at the sn-1 position and are thus sub-classified into alkylacyl phospholipids. Furthermore, as shown in Figure 5B, Cer-NS (d18:1/16:0), Cer-NS (d18:1/22:0), Cer-NS (d18:1/24:1), FA (22:5), PG (16:0-16:1), PS (18:0-20:3) exhibited suggestive correlation (edges ≥ 14). Indubitable, correlation between different classes of phospholipids existed. Additionally, polyunsaturated fatty acids showed more correlation with ceramide non hydroxyfatty acid-sphingosine (Cer-NS). Although the relationship between FA and Cer remains unclear in pulmonary disease, C16:0 ceramide has been identified as the principal mediator of obesity-derived insulin resistance and impaired fatty acid oxidation (Raichur et al., 2014). The high level of correlation between the lipids implied that HL Granule may regulate lipid homeostasis by interaction between lipids.

## Discussion

HL Granule is composed of nine traditional Chinese medicines, and it has been shown that several herbs functions well in the treatment of acute asthma. Theoretically, *Ephedra sinica* is considered to be effective, which may have the effects of dispersing external wind and calming latent wind. It was found that Mahuang-Tang, Mahuang-Xixin-Fuzi decoction and Shegan-Mahuang Decoction, which are consisted of *Ephedra sinica* and *Belamcanda chinensis*, mitigated airway inflammation and asthmatic airway hyperresponsiveness (He et al., 2018; Gan et al., 2020; Lin et al., 2020). *Lepidium apetalum*, *Platycodon grandiflorum* and *Pheretima* were also found effective in asthma by alleviating airway inflammation (Huang et al., 2016; Kim et al., 2019a; Lee et al., 2020). Recent evidence suggested that formononetin and calycosin, two flavonoids predominantly representing in *Astragalus atropilosulus*, could alleviate allergic asthma by protecting epithelial integrity via regulating and activating G protein coupled estrogen receptor (Yuan et al., 2020). For supplementary therapy, the coadministration of budesonide and the extracts of *Epimedium brevicornu* in asthma were better than budesonide individual treatment (Ma et al., 2020). Thus, it is considerable to treat asthma with a combination consisting of HL Granule, which was confirmed by our experiments.

Th2 cell-mediated immunity has dominated in the past 30 years of research on the pathogenesis of asthma. IL-4, IL-5 and IL-13 are signature cytokines in Th2 cell mediated immunity. Clinically, serum IL-4, IL-5 and IL-13 levels increased significantly in patients with acute asthma (Lee et al., 2001). Thus, we combined pulmonary function, histopathology, IL-4, IL-5, and IL-13 to observe the therapeutic effect of HL Granule. The results suggested that HL Granule could improve lung function, reduce the inflammatory infiltration of lung tissue, especially around the airway, and decrease IL-4 and IL-5 expressions to relieve acute asthma, although airway remodeling was not obvious. However, some studies have shown that the thickness of airway smooth muscle, which is an important change in airway remodeling, is related to the severity of asthma, not to asthma progression (Hirota and Martin, 2013). Moreover, IL-13 induces excessive mucus production and goblet cell metaplasia (Kuperman et al., 2002). In our model, few mucus and goblet cells were observed by pathological results, which may be one of the reasons for no significant changes to IL-13.

Lipidomics is considered to be an indispensable tool for the researches of many diseases and physiological processes, and has been used to study inflammation-related diseases (Zhang et al., 2018). Clinical findings elucidated that lipid metabolism disorders are frequently observed in asthma patients (Kang et al., 2014; Gai et al., 2019). Therefore, regulating lipid homeostasis for asthma treatment is crux. Presently, there are few studies on the mechanism of regulating lipids on therapeutic drugs for asthma. In our study, we have studied the mechanism of HL Granule regulating lipid homeostasis.

Several studies show a significant treatment of asthma by components from traditional Chinese Medicine according to the regulation of lipid homeostasis. Metabolomics combined with network pharmacology clarified that *Astragalus atropilosulus* regulated arachidonic acid metabolism and ether lipid metabolism (Wang et al., 2019). *Platycodon grandiflorum* and *Crataegus pinnatifida* ethanol extracts, as well as *Ephedra sinica* methanol extracts may also improve lipid metabolism (Lee et al., 2017; Kim et al., 2019b; Lee et al., 2019). Remarkably, ephedrine, astragaloside IV, calycosin and icariin, which were detected and identified in present study, may be linked to lipids alterations. The chronic-effect study of ephedrine determined that brown adipose tissue (BAT) activity was significantly reduced after a 28 days ephedrine treatment (Carey et al., 2013; Carey et al., 2015). Studies in liver found that astragaloside IV attenuated lipid accumulation in an AMPK-dependent manner (Zhou et al., 2017), while hairy calycosin could effectively control the lipid peroxidation, reduce the levels of serum free fatty acid, and improve the steatosis (Liu et al., 2019). In addition, many studies have shown that the metabolites of icariin have a lipid-lowering effect, which has attracted widespread attention in recent years (Wang et al., 2020). Thus, HL Granule has advantages in regulating lipid homeostasis. However, further studies are needed to explore the detailed mechanism among them.

To explore the mechanism of HL Granule in regulating lipid homeostasis, we used high-resolution lipidomics analysis based on UHPLC-Q-Exactive Orbitrap MS to recognize potential disease-related lipid changes in lung tissue. The method allows to detect a total of 304 and 167 lipids in positive and negative ion mode, of which 162 and 109 lipids in the model group were upregulated (FDR < 0.05, Fold change >2 or Fold change <0.5). Among them, HL Granule could reverse 104 and 73 lipids with statistical difference (FDR < 0.05). In the lung lipidomics, we identified at least thirteen types of lipids are regulated by HL Granule, including ACar, FA, LPC, PC, LPE, PE, PG, PI, PS, DG, TG, SM, and Cer. The observation suggested that HL Granule improved lipid homeostasis of acute asthmatic. The study enables us to understand the new pathway and pharmacological mechanism of HL Granule in the pathogenesis of asthma mediated by pulmonary lipid disorders.

Lung tissue contains 3–4% lipids in wet weight, and about 60% of the lipids are phospholipids (Rouser et al., 1969; Toshima and Akino, 1972). Phospholipids are important parts of cell membrane structure, and some pulmonary phospholipids are the main components of pulmonary surfactant in alveoli. In present study, phospholipids were disordered with the largest number in the lung tissue of acute asthma. The comprehensive analysis of heredity and metabolites suggests that PC increased in patients with asthma (Ried et al., 2013), which is consistent with our results, and HL Granule could downregulate the levels of PC. PE synthesis is critical for mitochondrial and endoplasmic reticulum function. The increased PE levels observed in the model groups might be reflective of an increased mitochondrial energy production. This assumption would be further supported by the increased levels of acylcarnitine levels. Additionally, our work reveals the levels of ether phospholipids are increased significantly in the model groups. Ether phospholipids in the plasma membrane act as the substrates for the lipid peroxidation and resulting in the further cell death (Zou et al., 2020). The downregulation of PEe levels suggested the protective effects of HL Granule. It has long been known that ether-linked phospholipids are abundantly present in neutrophils (Nagan and Zoeller, 2001), which could be rapidly recruited and are largely present in the airways of allergic eosinophilic asthmatic patients (Radermecker et al., 2018). A recent data suggested PEe and PCe were associated with ferroptosis sensitivity, which was involved in various pulmonary diseases (Zou et al., 2020).

PG predominantly observed in the lung tissue. The increased levels of PGs might inhibit Toll-like receptor (TLR)-mediated inflammation, and improved mitochondrial activity and inflammation (Chen et al., 2018; Choudhary et al., 2019). In present study, HL Granule downregulated partial PG and PE levels, suggesting a protective of the OVA induced asthma. PIs are the ubiquitous component of eukaryotic cells that participate in signal processes. Especially, glycerophosphoinositol 4-phosphate controls actin dynamics in cell systems (Corda et al., 2002). The literature reported that the PI antagonized the activation of homologous ligands of TLR 2 and TLR 4, influencing innate immunity and the transcription of many pro-inflammatory genes (Voelker and Numata, 2019). PS is also one of the most abundant lipids in plasma membrane, and recent literatures reported that the exposed PS on the outer leaflet of the plasma membrane is the functional ligand for the signal pathway (Gong et al., 2017). Proteins interacting with PS are involved in almost all aspects of cellular regulation by activating protein kinases and transcription. Phagocytes recognized PS on apoptotic cells which were then cleared through CD36 and oxLPL receptors (Zhang et al., 2018). In the experimental model of allergic airway inflammation, apoptotic cells and inflammatory dendritic cells that express PS inhibitory immune receptor CD300a increased significantly after intraperitoneal injection of alum combined with OVA (Miki et al., 2015). It could be the reason for the increase of PS in the model group, and HL Granule might affect PS related proteins or cells.

Lysophospholipids (LPs) are produced by hydrolysis of phospholipids according to phospholipase A2 (PLA2). LPs exist in all types of cells, as a second messenger molecule to regulate intracellular signaling pathways. LPs also participate in many biological functions, including inflammation (Arifin and Falasca, 2016). In present study, we mainly changed LPC and LPE. Although some studies showed that some LPCs decreased in fasting serum (Ried et al., 2013). However, increasing level of LPC usually played a pathogenic role in the inflammatory injury of asthma (Bansal et al., 2016). LPC also induced migration of lymphocytes and macrophages, inhibited activation and migration of eosinophils, increased production of pro-inflammatory cytokines, aggravated oxidative stress and promotes apoptosis, thus accumulating inflammation and promoting the development of diseases (Knuplez et al., 2020; Liu et al., 2020). In the study, the upregulated LPCs were recalled by HL Granule which may be one of the reasons for its improvement of inflammation in acute asthma.

Sphingolipid serve as receptors for multiple pathogens and play key roles in immune signaling. Several studies reported that the asthma associated ORMDL3 (ORMDL Sphingolipid Biosynthesis Regulator 3) gene regulated the sphingolipid biosynthesis, and the altered sphingolipids modulate the T cells’ metabolism. Our data revealed significant correlation between the ceramide non-hydroxyfatty acid sphingosine (Cer-NS) and long chain fatty acids, suggested the sphingolipid metabolism was involved in the therapeutic action of HL Granule. The accumulation of ceramides is reported to be associated with apoptosis (Hannun and Obeid, 2018). The decreased ceramides of HL Granule suggested the possible anti-apoptosis effect. Moreover, the produced SMs from ceramides on the plasma are supposed to be an acute response to extracellular stimuli. Additionally, plasma ultra-long-chain SM (d18:1/24:0) might work for regulating the activation of macrophages and inflammatory response (Sakamoto et al., 2017).

A research showed that long-chain polyunsaturated fatty acids could be converted into lipid mediators during inflammation to modulate bronchoconstriction and airway inflammation (Fussbroich et al., 2019). The key enzymes of fatty acid oxidation are involved in the OVA induced asthma, which could significantly reduce allergen-induced AHR, the number of inflammatory cells, and the production of asthma-related cytokines and chemokines (Al-Khami et al., 2017). HL Granule could downregulate the levels of long-chain fatty acids, and further studies are required to identify the specific mechanisms. Correlation analysis revealed that the alteration of long chain fatty acids is highly correlated with that of ceramide levels. The results suggested the biosynthesis of ceramide from the fatty acids was involved in the protection of asthma by HL Granule. In addition, a specific ceramide species, C16:0 ceramide was recently identified regulating FAO and impairing fatty acid oxidation in obesity (Raichur et al., 2014). Our results show that HL Granule downregulated the level of acyl carnitine, which has the potential to activate inflammation. We also identified that DG and TG increased uniformly in the lung tissue, and HL Granule could regulate these upregulated lipids.

In summary, our research confirmed that HL Granule could be used in acute asthma treatment, but one deserved attention, is that different doses of HL Granule in the treatment, including variable treatment dose, optimal treatment dose, toxicities underwent dose, and so on, should be focused on in next studies. Additionally, there are few studies on lung lipids and asthma at present, and our exploration of the effect of HL Granule on acute asthma by regulating pulmonary lipid homeostasis is also preliminary. Further researches are needed to verify and reveal the role and interaction of lipids in asthma and drug intervention.

## Conclusion

In conclusion, we established an acute model of asthma with AHR, airway inflammation, and increasing levels of IL-4 and IL-5, which could be effectively reduced by the treatment of HL Granule. A total of 304 and 167 lipids were detected and identified in positive and negative ion mode in lung tissue, of which 162 and 109 lipids were significantly upregulated in model group (FDR < 0.05, FC > 2 or FC < 0.5). 104 and 73 lipids could be reversed by HL Granule, with statistical difference (FDR < 0.05), including ACar, FA, LPC, PC, LPE, PE, PG, PI, PS, DG, TG, SM, and Cer. Notably, 118 and 273 correlations among 47 and 96 lipids in the positive and negative were observed, with PEe and PCe (FDR < 0.001, Spearman correlation coefficient r^2^ > 0.75). Therefore, we found that lipid disorders play an important role in asthma. HL Granule may regulate pulmonary lipid homeostasis for the treatment of acute asthma. Furthermore, our study has significance for clinical guides. In view of the side effects of drugs used in the treatment of asthma such as montelukast sodium, HL Granule could be used as an alternative or supplementary therapy in clinic.

## Data Availability

The raw data supporting the conclusion of this article will be made available by the authors, without undue reservation, to any qualified researcher.

## Abbreviations

ACar, acylcarnitine; ACar, acylcarnitine; AHR, airway hyperresponsiveness; BALF, bronchoalveolar lavage fluid; Cer, ceramide; DG, diglyceride; ELISA, Enzyme linked immunosorbent assay; FA, fatty acid; HL Granule, Huanglong antitussive granule; LC-MS, liquid chromatography-mass spectrometry; LPC, lysophosphatidylcholine; LPE, lysophosphatidylethanolamine; OVA, ovalbumin; PC, phosphatidylcholine; PCe, ether-linked phosphatidylcholine; PE, phosphatidylethanolamine; PEe, ether-linked phosphatidylethanolamine; Penh, enhance pause; PG, phosphatidylglycerol; PI, phosphatidylinositol; PS, phosphatidylserine; SM, sphingomyelin; TG, triglyceride; TIC, total ion chromatograms; UHPLC-Q-Exactive Orbitrap MS, ultra-high-performance liquid chromatography coupled to hybrid Quadrupole-Exactive Orbitrap mass spectrometry
