# Supplementary material for: Huanglong Antitussive Granule Relieves Acute Asthma Through Regulating Pulmonary Lipid Homeostasis
Source: Front Pharmacol. 2021 Apr 23;12:656756. doi: 10.3389/fphar.2021.656756 (PMC8103164; doi:10.3389/fphar.2021.656756)
Supplement: Supplementary file 1 [file DataSheet1.docx]

**Table S1.** Description of HL Granule

| **Formulation** | **Species** | **Family** | **Part** | **Weight (g)** |
| --- | --- | --- | --- | --- |
| HL Granule | Astragalus atropilosulus (Hochst.) Bunge (Huangqi) | Leguminosae | root | 0.27 |
|  | Epimedium brevicornu Maxim. (Yinyanghuo) | Berberidaceae | leaf | 0.30 |
|  | Platycodon grandiflorum (Jacq.) A.DC. (Jiegeng) | Campanulaceae | root | 0.09 |
|  | Pheretima aspergillum (Dilong) | Megascolecidae | body | 0.09 |
|  | Belamcanda chinensis (L.) Redouté (Shegan) | Iridaceae | rhizome | 0.18 |
|  | Houttuynia cordata Thunb. (Yuxingcao) | Saururaceae | overground | 0.60 |
|  | Ephedra sinica Stapf (Mahuang) | Ephedraceae | stem | 0.09 |
|  | Crataegus pinnatifida Bunge (Shanzha) | Rosaceae | fruit | 0.45 |
|  | Lepidium apetalum Willd. (Tinglizi) | Cruciferae | seed | 0.18 |

The extraction of one packet contains the species section.

**Table S2.** Different lipids in lung (positive ion mode)

| **Significant metabolites** | **m/z** | **t_R_(min)** | **Adduct ion** | **Kruskal-Wallis** | **Model/Control(Mann-Whitney)** | | | **HL Granule/Model(Mann-Whitney)** | | |
| --- | --- | --- | --- | --- | --- | --- | --- | --- | --- | --- |
|  |  |  |  |  | **p value** | **FDR** | **Fold Change** | **p value** | **FDR** | **Fold Change** |
| ACar 16:0 | 400.3409 | 3.2 | [M]+ | 0.0111 | 0.0087^##^ | 0.0165^#^ | 2.34 | 0.0087^**^ | 0.0286^*^ | 0.44 |
| LPC 18:0e | 510.3925 | 5.31 | [M+H]+ | 0.0029 | 0.0022^##^ | 0.0049^##^ | 2.05 | 0.0152^*^ | 0.0397^*^ | 0.65 |
| LPC 20:0(SN1) | 552.4071 | 6.52 | [M+H]+ | 0.0009 | 0.0022^##^ | 0.0049^##^ | 2.42 | 0.0087^**^ | 0.0286^*^ | 0.69 |
| LPC 20:1(SN1) | 550.3849 | 4.82 | [M+H]+ | 0.0029 | 0.0022^##^ | 0.0049^##^ | 2.40 | 0.0152^*^ | 0.0397^*^ | 0.61 |
| LPC 22:6(SN2) | 568.3422 | 2.1 | [M+H]+ | 0.0115 | 0.0087^##^ | 0.0165^#^ | 2.07 | 0.0152^*^ | 0.0397^*^ | 0.62 |
| LPC 24:0(SN1) | 608.4667 | 9.27 | [M+H]+ | 0.0012 | 0.0022^##^ | 0.0049^##^ | 2.06 | 0.0087^**^ | 0.0286^*^ | 0.67 |
| PC 12:0-18:1 | 726.5034 | 9.86 | [M+Na]+ | 0.0039 | 0.0022^##^ | 0.0049^##^ | 2.48 | 0.0152^*^ | 0.0397^*^ | 0.58 |
| PC 13:1-21:2 | 778.5391 | 10.27 | [M+Na]+ | 0.0021 | 0.0022^##^ | 0.0049^##^ | 2.93 | 0.0152^*^ | 0.0397^*^ | 0.54 |
| PC 14:0-14:0 | 678.5088 | 9.68 | [M+H]+ | 0.0021 | 0.0022^##^ | 0.0049^##^ | 2.15 | 0.0152^*^ | 0.0397^*^ | 0.74 |
| PC 14:0-18:3 | 750.5005 | 9.89 | [M+Na]+ | 0.0055 | 0.0043^##^ | 0.0088^##^ | 3.28 | 0.0152^*^ | 0.0397^*^ | 0.54 |
| PC 14:1-22:4 | 802.5326 | 10.12 | [M+Na]+ | 0.0014 | 0.0022^##^ | 0.0049^##^ | 2.59 | 0.0087^**^ | 0.0286^*^ | 0.56 |
| PC 16:0-16:0 | 734.564 | 12.5 | [M+H]+ | 0.0017 | 0.0022^##^ | 0.0049^##^ | 2.25 | 0.0152^*^ | 0.0397^*^ | 0.66 |
| PC 16:0-18:1 | 760.5834 | 12.11 | [M+H]+ | 0.0011 | 0.0022^##^ | 0.0049^##^ | 2.27 | 0.0043^**^ | 0.0196^*^ | 0.68 |
| PC 16:0-18:2 | 758.5684 | 11.21 | [M+H]+ | 0.0017 | 0.0022^##^ | 0.0049^##^ | 2.19 | 0.0152^*^ | 0.0397^*^ | 0.69 |
| PC 16:0-18:2 | 780.5519 | 11.21 | [M+Na]+ | 0.0017 | 0.0022^##^ | 0.0049^##^ | 2.14 | 0.0152^*^ | 0.0397^*^ | 0.72 |
| PC 16:0-20:4 | 782.5723 | 11.06 | [M+H]+ | 0.0009 | 0.0022^##^ | 0.0049^##^ | 2.65 | 0.0022^**^ | 0.0143^*^ | 0.63 |
| PC 16:0-22:5 | 830.5659 | 11.12 | [M+Na]+ | 0.0006 | 0.0022^##^ | 0.0049^##^ | 3.36 | 0.0022^**^ | 0.0143^*^ | 0.63 |
| PC 14:1-22:2 | 806.5705 | 11.35 | [M+Na]+ | 0.0033 | 0.0043^##^ | 0.0088^##^ | 2.54 | 0.0022^**^ | 0.0143^*^ | 0.61 |
| PC 16:1-16:1 | 730.5406 | 10.09 | [M+H]+ | 0.0025 | 0.0022^##^ | 0.0049^##^ | 2.25 | 0.0152^*^ | 0.0397^*^ | 0.70 |
| PC 18:0-18:1 | 810.5948 | 13.36 | [M+Na]+ | 0.0021 | 0.0022^##^ | 0.0049^##^ | 2.26 | 0.0022^**^ | 0.0143^*^ | 0.60 |
| PC 18:0-18:2 | 808.584 | 12.52 | [M+Na]+ | 0.0011 | 0.0022^##^ | 0.0049^##^ | 3.78 | 0.0022^**^ | 0.0143^*^ | 0.44 |
| PC 18:0-20:5 | 830.5679 | 11.48 | [M+Na]+ | 0.0008 | 0.0022^##^ | 0.0049^##^ | 4.71 | 0.0022^**^ | 0.0143^*^ | 0.46 |
| PC 18:1-22:6 | 854.5674 | 10.9 | [M+Na]+ | 0.0017 | 0.0022^##^ | 0.0049^##^ | 3.45 | 0.0152^*^ | 0.0397^*^ | 0.63 |
| PC 18:2-18:2 | 804.5544 | 10.52 | [M+Na]+ | 0.0012 | 0.0022^##^ | 0.0049^##^ | 2.35 | 0.0022^**^ | 0.0143^*^ | 0.47 |
| PC 18:2-18:2 | 782.5722 | 10.51 | [M+H]+ | 0.0012 | 0.0022^##^ | 0.0049^##^ | 2.69 | 0.0087^**^ | 0.0286^*^ | 0.62 |
| PC 18:2-20:4 | 828.549 | 10.36 | [M+Na]+ | 0.0008 | 0.0022^##^ | 0.0049^##^ | 2.35 | 0.0087^**^ | 0.0286^*^ | 0.60 |
| PC 18:2-22:3 | 858.5984 | 12.29 | [M+Na]+ | 0.0006 | 0.0022^##^ | 0.0049^##^ | 2.69 | 0.0022^**^ | 0.0143^*^ | 0.57 |
| PC 20:3-20:3 | 856.5798 | 11.51 | [M+Na]+ | 0.0012 | 0.0022^##^ | 0.0049^##^ | 4.40 | 0.0087^**^ | 0.0286^*^ | 0.63 |
| PC 14:0e/16:0 | 692.56 | 11.46 | [M+H]+ | 0.0009 | 0.0022^##^ | 0.0049^##^ | 2.18 | 0.0022^**^ | 0.0143^*^ | 0.72 |
| PC 14:0e/18:1 | 718.575 | 12.53 | [M+H]+ | 0.0009 | 0.0022^##^ | 0.0049^##^ | 2.76 | 0.0043^**^ | 0.0196^*^ | 0.62 |
| PC 14:0e/20:2 | 744.5922 | 12.67 | [M+H]+ | 0.0011 | 0.0022^##^ | 0.0049^##^ | 2.50 | 0.0043^**^ | 0.0196^*^ | 0.63 |
| PC 14:0e/22:3 | 770.6089 | 12.25 | [M+H]+ | 0.0008 | 0.0022^##^ | 0.0049^##^ | 2.08 | 0.0022^**^ | 0.0143^*^ | 0.64 |
| PC 14:0e/22:5 | 766.5757 | 11.53 | [M+H]+ | 0.0014 | 0.0022^##^ | 0.0049^##^ | 2.93 | 0.0043^**^ | 0.0196^*^ | 0.58 |
| PC 14:0e/24:4 | 796.6219 | 12.63 | [M+H]+ | 0.0009 | 0.0022^##^ | 0.0049^##^ | 2.02 | 0.0022^**^ | 0.0143^*^ | 0.68 |
| PC 14:1e/4:0 | 522.3541 | 3.09 | [M+H]+ | 0.0044 | 0.0022^##^ | 0.0049^##^ | 2.24 | 0.0152^*^ | 0.0397^*^ | 0.62 |
| PC 16:0e/22:6 | 792.5919 | 11.53 | [M+H]+ | 0.0005 | 0.0022^##^ | 0.0049^##^ | 3.18 | 0.0022^**^ | 0.0143^*^ | 0.57 |
| PC 16:2e/22:6 | 788.554 | 11.53 | [M+H]+ | 0.0008 | 0.0022^##^ | 0.0049^##^ | 4.03 | 0.0043^**^ | 0.0196^*^ | 0.60 |
| PC 16:2e/24:4 | 820.6215 | 11.9 | [M+H]+ | 0.0011 | 0.0022^##^ | 0.0049^##^ | 2.46 | 0.0043^**^ | 0.0196^*^ | 0.61 |
| PC 18:1e/22:6 | 818.6083 | 11.57 | [M+H]+ | 0.0021 | 0.0022^##^ | 0.0049^##^ | 3.25 | 0.0022^**^ | 0.0143^*^ | 0.50 |
| PC 18:2e/22:6 | 816.5909 | 11.81 | [M+H]+ | 0.0005 | 0.0022^##^ | 0.0049^##^ | 2.98 | 0.0022^**^ | 0.0143^*^ | 0.52 |
| PC 18:4e/22:6 | 812.5583 | 11.21 | [M+H]+ | 0.0008 | 0.0022^##^ | 0.0049^##^ | 2.80 | 0.0043^**^ | 0.0196^*^ | 0.63 |
| LPE 16:1e | 438.2989 | 3.29 | [M+H]+ | 0.0009 | 0.0022^##^ | 0.0049^##^ | 2.34 | 0.0043^**^ | 0.0196^*^ | 0.65 |
| LPE 18:1e | 466.33 | 5.28 | [M+H]+ | 0.0008 | 0.0022^##^ | 0.0049^##^ | 2.80 | 0.0022^**^ | 0.0143^*^ | 0.61 |
| PE 16:0-16:0 | 692.5231 | 12.25 | [M+H]+ | 0.0006 | 0.0022^##^ | 0.0049^##^ | 2.80 | 0.0022^**^ | 0.0143^*^ | 0.60 |
| PE 16:0-18:1 | 718.5375 | 12.41 | [M+H]+ | 0.0009 | 0.0022^##^ | 0.0049^##^ | 2.55 | 0.0152^*^ | 0.0397^*^ | 0.68 |
| PE 16:0-18:2 | 716.5173 | 11.49 | [M+H]+ | 0.0008 | 0.0022^##^ | 0.0049^##^ | 2.66 | 0.0022^**^ | 0.0143^*^ | 0.61 |
| PE 16:0-22:5 | 766.5334 | 11.45 | [M+H]+ | 0.0006 | 0.0022^##^ | 0.0049^##^ | 2.46 | 0.0022^**^ | 0.0143^*^ | 0.65 |
| PE 16:1-18:1 | 716.5219 | 11.37 | [M+H]+ | 0.0006 | 0.0022^##^ | 0.0049^##^ | 2.85 | 0.0043^**^ | 0.0196^*^ | 0.60 |
| PE 16:2-22:5 | 762.5059 | 11.32 | [M+H]+ | 0.0006 | 0.0022^##^ | 0.0049^##^ | 2.17 | 0.0022^**^ | 0.0143^*^ | 0.71 |
| PE 18:0-18:1 | 746.5671 | 13.69 | [M+H]+ | 0.0012 | 0.0022^##^ | 0.0049^##^ | 2.91 | 0.0087^**^ | 0.0286^*^ | 0.60 |
| PE 18:0-18:2 | 744.5501 | 12.73 | [M+H]+ | 0.0009 | 0.0022^##^ | 0.0049^##^ | 2.71 | 0.0043^**^ | 0.0196^*^ | 0.61 |
| PE 18:0-20:3 | 770.5664 | 13.05 | [M+H]+ | 0.0012 | 0.0022^##^ | 0.0049^##^ | 2.42 | 0.0087^**^ | 0.0286^*^ | 0.64 |
| PE 18:0-20:4 | 768.5526 | 12.54 | [M+H]+ | 0.0005 | 0.0022^##^ | 0.0049^##^ | 2.60 | 0.0022^**^ | 0.0143^*^ | 0.56 |
| PE 18:0-22:6 | 792.5496 | 12.21 | [M+H]+ | 0.0006 | 0.0022^##^ | 0.0049^##^ | 2.78 | 0.0022^**^ | 0.0143^*^ | 0.69 |
| PE 18:1-18:1 | 744.5463 | 12.56 | [M+H]+ | 0.0006 | 0.0022^##^ | 0.0049^##^ | 2.72 | 0.0043^**^ | 0.0196^*^ | 0.63 |
| PE 18:1-18:2 | 742.5386 | 11.63 | [M+H]+ | 0.0005 | 0.0022^##^ | 0.0049^##^ | 2.32 | 0.0022^**^ | 0.0143^*^ | 0.65 |
| PE 20:2-20:2 | 796.5852 | 13.43 | [M+H]+ | 0.0011 | 0.0022^##^ | 0.0049^##^ | 2.54 | 0.0022^**^ | 0.0143^*^ | 0.63 |
| PE 16:0e/16:1 | 676.5268 | 12.89 | [M+H]+ | 0.0014 | 0.0022^##^ | 0.0049^##^ | 2.85 | 0.0152^*^ | 0.0397^*^ | 0.56 |
| PE 16:0e/16:2 | 674.5127 | 11.79 | [M+H]+ | 0.0006 | 0.0022^##^ | 0.0049^##^ | 2.76 | 0.0043^**^ | 0.0196^*^ | 0.60 |
| PE 16:0e/18:3 | 700.5272 | 12.06 | [M+H]+ | 0.0011 | 0.0022^##^ | 0.0049^##^ | 2.52 | 0.0043^**^ | 0.0196^*^ | 0.59 |
| PE 16:0e/20:2 | 730.5709 | 14.32 | [M+H]+ | 0.0005 | 0.0022^##^ | 0.0049^##^ | 2.82 | 0.0022^**^ | 0.0143^*^ | 0.64 |
| PE 16:0e/20:3 | 728.5602 | 13.18 | [M+H]+ | 0.0011 | 0.0022^##^ | 0.0049^##^ | 3.13 | 0.0022^**^ | 0.0143^*^ | 0.56 |
| PE 16:0e/20:4 | 726.5416 | 12.39 | [M+H]+ | 0.0009 | 0.0022^##^ | 0.0049^##^ | 2.51 | 0.0022^**^ | 0.0143^*^ | 0.62 |
| PE 16:0e/20:5 | 724.5298 | 11.86 | [M+H]+ | 0.0008 | 0.0022^##^ | 0.0049^##^ | 2.61 | 0.0043^**^ | 0.0196^*^ | 0.64 |
| PE 16:0e/22:6 | 750.5391 | 11.74 | [M+H]+ | 0.0006 | 0.0022^##^ | 0.0049^##^ | 2.40 | 0.0043^**^ | 0.0196^*^ | 0.59 |
| PE 18:0e/20:5 | 752.5616 | 13.11 | [M+H]+ | 0.0005 | 0.0022^##^ | 0.0049^##^ | 2.34 | 0.0022^**^ | 0.0143^*^ | 0.69 |
| PE 18:0e/22:6 | 778.5711 | 13.16 | [M+H]+ | 0.0017 | 0.0022^##^ | 0.0049^##^ | 2.49 | 0.0087^**^ | 0.0286^*^ | 0.58 |
| PE 18:1e/16:1 | 702.5441 | 13.03 | [M+H]+ | 0.0005 | 0.0022^##^ | 0.0049^##^ | 2.69 | 0.0022^**^ | 0.0143^*^ | 0.61 |
| PE 18:1e/20:2 | 756.5925 | 14.38 | [M+H]+ | 0.0009 | 0.0022^##^ | 0.0049^##^ | 2.85 | 0.0022^**^ | 0.0143^*^ | 0.66 |
| PE 18:1e/20:3 | 754.5728 | 13.33 | [M+H]+ | 0.0005 | 0.0022^##^ | 0.0049^##^ | 2.51 | 0.0022^**^ | 0.0143^*^ | 0.64 |
| PE 18:1e/20:4 | 752.5596 | 12.51 | [M+H]+ | 0.0005 | 0.0022^##^ | 0.0049^##^ | 3.15 | 0.0022^**^ | 0.0143^*^ | 0.53 |
| PE 18:1e/20:5 | 750.5388 | 11.93 | [M+H]+ | 0.0006 | 0.0022^##^ | 0.0049^##^ | 2.42 | 0.0022^**^ | 0.0143^*^ | 0.63 |
| PE 18:1e/22:5 | 778.5706 | 12.92 | [M+H]+ | 0.0009 | 0.0022^##^ | 0.0049^##^ | 2.17 | 0.0022^**^ | 0.0143^*^ | 0.65 |
| PE 18:1e/22:6 | 776.5554 | 12.06 | [M+H]+ | 0.0006 | 0.0022^##^ | 0.0049^##^ | 2.85 | 0.0022^**^ | 0.0143^*^ | 0.64 |
| PE 18:2e/18:2 | 726.5417 | 12.12 | [M+H]+ | 0.0008 | 0.0022^##^ | 0.0049^##^ | 2.90 | 0.0022^**^ | 0.0143^*^ | 0.53 |
| PE 18:2e/18:3 | 724.5308 | 11.28 | [M+H]+ | 0.0005 | 0.0022^##^ | 0.0049^##^ | 2.49 | 0.0022^**^ | 0.0143^*^ | 0.65 |
| PG 18:0-22:6 | 840.5781 | 10.85 | [M+NH4]+ | 0.0057 | 0.0022^##^ | 0.0049^##^ | 4.80 | 0.0087^**^ | 0.0286^*^ | 0.17 |
| DAG 16:0-20:3 | 636.5591 | 14.18 | [M+NH4]+ | 0.0014 | 0.0022^##^ | 0.0049^##^ | 2.95 | 0.0043^**^ | 0.0196^*^ | 0.58 |
| DAG 16:0-20:4 | 634.5417 | 13.56 | [M+NH4]+ | 0.0014 | 0.0022^##^ | 0.0049^##^ | 3.39 | 0.0087^**^ | 0.0286^*^ | 0.56 |
| DAG 16:0-22:5 | 660.5535 | 13.63 | [M+NH4]+ | 0.0009 | 0.0022^##^ | 0.0049^##^ | 3.10 | 0.0087^**^ | 0.0286^*^ | 0.57 |
| DAG 18:0-20:3 | 664.5891 | 15.44 | [M+NH4]+ | 0.0017 | 0.0022^##^ | 0.0049^##^ | 2.92 | 0.0087^**^ | 0.0286^*^ | 0.66 |
| DAG 18:0-20:4 | 662.5707 | 14.87 | [M+NH4]+ | 0.0011 | 0.0022^##^ | 0.0049^##^ | 3.05 | 0.0043^**^ | 0.0196^*^ | 0.60 |
| TAG 16:0-18:0-22:0 | 936.8951 | 25.22 | [M+NH4]+ | 0.0115 | 0.0260 | 0.0441 | 2.30 | 0.0152^*^ | 0.0397^*^ | 0.63 |
| TAG 16:0-22:6-22:6 | 973.7195 | 20.02 | [M+Na]+ | 0.0091 | 0.0087^##^ | 0.0165^#^ | 2.30 | 0.0087^**^ | 0.0286^*^ | 0.63 |
| TAG 16:1-16:1-18:1 | 851.709 | 21.46 | [M+Na]+ | 0.0028 | 0.0022^##^ | 0.0049^##^ | 3.63 | 0.0022^**^ | 0.0143^*^ | 0.55 |
| SM d14:0/18:1 | 675.5433 | 9.42 | [M+H]+ | 0.0009 | 0.0022^##^ | 0.0049^##^ | 2.26 | 0.0022^**^ | 0.0143^*^ | 0.68 |
| SM d14:0/19:1 | 689.5618 | 9.98 | [M+H]+ | 0.0008 | 0.0022^##^ | 0.0049^##^ | 2.27 | 0.0043^**^ | 0.0196^*^ | 0.70 |
| SM d14:0/20:0 | 705.5917 | 10.98 | [M+H]+ | 0.0005 | 0.0022^##^ | 0.0049^##^ | 2.19 | 0.0022^**^ | 0.0143^*^ | 0.66 |
| SM d14:0/22:1 | 731.6052 | 11.78 | [M+H]+ | 0.0011 | 0.0022^##^ | 0.0049^##^ | 2.26 | 0.0022^**^ | 0.0143^*^ | 0.68 |
| SM d14:0/24:1 | 759.6395 | 13.02 | [M+H]+ | 0.0031 | 0.0022^##^ | 0.0049^##^ | 2.06 | 0.0087^**^ | 0.0286^*^ | 0.79 |
| SM d14:0/28:2 | 813.6883 | 14.31 | [M+H]+ | 0.0017 | 0.0022^##^ | 0.0049^##^ | 2.58 | 0.0022^**^ | 0.0143^*^ | 0.57 |
| SM d14:0/30:2 | 841.7203 | 15.63 | [M+H]+ | 0.0024 | 0.0022^##^ | 0.0049^##^ | 2.70 | 0.0043^**^ | 0.0196^*^ | 0.56 |
| SM d14:1/28:2 | 811.668 | 13.28 | [M+H]+ | 0.0011 | 0.0022^##^ | 0.0049^##^ | 2.22 | 0.0022^**^ | 0.0143^*^ | 0.59 |
| SM d14:1/29:1 | 827.702 | 15 | [M+H]+ | 0.0011 | 0.0022^##^ | 0.0049^##^ | 2.16 | 0.0022^**^ | 0.0143^*^ | 0.71 |
| Cer-NS d18:1/20:0 | 594.5819 | 14.93 | [M+H]+ | 0.0006 | 0.0022^##^ | 0.0049^##^ | 2.61 | 0.0022^**^ | 0.0143^*^ | 0.64 |
| Cer-NS d18:1/24:0 | 650.644 | 17.5 | [M+H]+ | 0.0017 | 0.0022^##^ | 0.0049^##^ | 3.08 | 0.0152^*^ | 0.0397^*^ | 0.52 |
| Cer-NS d18:2/18:0 | 564.5334 | 12.5 | [M+H]+ | 0.0014 | 0.0022^##^ | 0.0049^##^ | 3.69 | 0.0087^**^ | 0.0286^*^ | 0.50 |
| Cer-NS d18:2/20:0 | 592.5662 | 13.82 | [M+H]+ | 0.0029 | 0.0022^##^ | 0.0049^##^ | 3.03 | 0.0152^*^ | 0.0397^*^ | 0.60 |
| Cer-NS d18:2/22:1 | 618.5836 | 13.86 | [M+H]+ | 0.0025 | 0.0022^##^ | 0.0049^##^ | 3.11 | 0.0152^*^ | 0.0397^*^ | 0.51 |
| HexCer-NS d18:1/16:0 | 700.5713 | 11.08 | [M+H]+ | 0.0008 | 0.0022^##^ | 0.0049^##^ | 2.70 | 0.0087^**^ | 0.0286^*^ | 0.66 |
| HexCer-NS d18:1/18:0 | 728.6071 | 12.32 | [M+H]+ | 0.0044 | 0.0022^##^ | 0.0049^##^ | 2.33 | 0.0152^*^ | 0.0397^*^ | 0.63 |
| HexCer-NS d18:1/22:0 | 784.6635 | 14.91 | [M+H]+ | 0.0012 | 0.0022^##^ | 0.0049^##^ | 2.19 | 0.0087^**^ | 0.0286^*^ | 0.68 |
| HexCer-NS d18:1/24:0 | 812.6992 | 16.18 | [M+H]+ | 0.0014 | 0.0022^##^ | 0.0049^##^ | 2.78 | 0.0043^**^ | 0.0196^*^ | 0.56 |
| HexCer-NS d18:1/24:1 | 810.6818 | 14.88 | [M+H]+ | 0.0014 | 0.0022^##^ | 0.0049^##^ | 2.03 | 0.0087^**^ | 0.0286^*^ | 0.66 |

Model/Control, ^#^ P<0.05; ^##^ P<0.01; HL Granule/Model, ^*^ P<0.05, ^**^ P<0.01; FDR: FDR-adjusted p value; The differential lipids are ranked by Fold Change.

**Table S3.** Different lipids in lung (negative ion mode)

| **Significant metabolites** | **m/z** | **t_R_(min)** | **Adduct ion** | **Kruskal-Wallis** | **Model/Control(Mann-Whitney)** | | | **HL Granule/Model(Mann-Whitney)** | | | |
| --- | --- | --- | --- | --- | --- | --- | --- | --- | --- | --- | --- |
|  |  |  |  |  | **p value** | **FDR** | **Fold Change** | **p value** | **FDR** | **Fold Change** | |
| FA 20:3 | 305.2472 | 4.87 | [M-H]- | 0.0017 | 0.0022^##^ | 0.0030^##^ | 2.59 | 0.0260^*^ | 0.0438^*^ | | 0.61 |
| FA 22:1 | 337.3109 | 8.86 | [M-H]- | 0.0014 | 0.0022^##^ | 0.0030^##^ | 2.25 | 0.0260^*^ | 0.0438^*^ | | 0.68 |
| FA 22:2 | 335.2945 | 7.95 | [M-H]- | 0.0021 | 0.0022^##^ | 0.0030^##^ | 2.71 | 0.0152^*^ | 0.0298^*^ | | 0.56 |
| FA 22:3 | 333.2789 | 6.9 | [M-H]- | 0.0021 | 0.0022^##^ | 0.0030^##^ | 2.73 | 0.0260^*^ | 0.0438^*^ | | 0.60 |
| FAHFA 18:1/22:3 | 613.5215 | 5.65 | [M-H]- | 0.0087 | 0.0043^##^ | 0.0056^##^ | 2.75 | 0.0260^*^ | 0.0438^*^ | | 0.49 |
| LPC 18:1 | 580.3611 | 3.08 | [M+Hac-H]- | 0.0048 | 0.0022^##^ | 0.0030^##^ | 2.16 | 0.0260^*^ | 0.0438^*^ | | 0.70 |
| PC 16:0e/22:6 | 804.6101 | 12.78 | [M+Hac-H]- | 0.0014 | 0.0022^##^ | 0.0030^##^ | 2.04 | 0.0152^*^ | 0.0298^*^ | | 0.77 |
| PC 16:1e/20:4 | 824.5846 | 11.48 | [M+Hac-H]- | 0.0013 | 0.0022^##^ | 0.0030^##^ | 2.10 | 0.0022^**^ | 0.0086^**^ | | 0.69 |
| LPE 16:0 | 452.276 | 2.77 | [M-H]- | 0.0008 | 0.0022^##^ | 0.0030^##^ | 3.22 | 0.0087^**^ | 0.0198^*^ | | 0.66 |
| LPE 18:0 | 480.3067 | 4.5 | [M-H]- | 0.0012 | 0.0022^##^ | 0.0030^##^ | 2.78 | 0.0087^**^ | 0.0198^*^ | | 0.57 |
| LPE 18:1 | 478.2915 | 3.12 | [M-H]- | 0.0006 | 0.0022^##^ | 0.0030^##^ | 2.15 | 0.0043^**^ | 0.0117^*^ | | 0.73 |
| LPE 16:1e | 436.2837 | 3.24 | [M-H]- | 0.0008 | 0.0022^##^ | 0.0030^##^ | 2.51 | 0.0043^**^ | 0.0117^*^ | | 0.60 |
| LPE 18:1e | 464.314 | 5.21 | [M-H]- | 0.0008 | 0.0022^##^ | 0.0030^##^ | 2.75 | 0.0022^**^ | 0.0086^**^ | | 0.62 |
| LPE 18:2e | 462.2971 | 3.63 | [M-H]- | 0.0008 | 0.0022^##^ | 0.0030^##^ | 2.80 | 0.0043^**^ | 0.0117^*^ | | 0.64 |
| PE 16:0-16:0 | 688.4926 | 11.13 | [M-H]- | 0.0005 | 0.0022^##^ | 0.0030^##^ | 2.12 | 0.0022^**^ | 0.0086^**^ | | 0.65 |
| PE 16:0-16:1 | 674.5144 | 12.77 | [M-H]- | 0.0017 | 0.0022^##^ | 0.0030^##^ | 2.59 | 0.0260^*^ | 0.0438^*^ | | 0.64 |
| PE 16:0-18:1 | 672.4998 | 11.75 | [M-H]- | 0.0006 | 0.0022^##^ | 0.0030^##^ | 2.35 | 0.0043^**^ | 0.0117^*^ | | 0.68 |
| PE 16:0-18:2 | 716.5201 | 12.31 | [M-H]- | 0.0009 | 0.0022^##^ | 0.0030^##^ | 2.18 | 0.0043^**^ | 0.0117^*^ | | 0.66 |
| PE 16:0-20:5 | 714.5055 | 11.28 | [M-H]- | 0.0017 | 0.0022^##^ | 0.0030^##^ | 2.42 | 0.0260^*^ | 0.0438^*^ | | 0.81 |
| PE 16:0-22:5 | 712.4902 | 10.43 | [M-H]- | 0.0008 | 0.0022^##^ | 0.0030^##^ | 2.70 | 0.0043^**^ | 0.0117^*^ | | 0.58 |
| PE 16:0-22:6 | 698.5128 | 11.96 | [M-H]- | 0.0008 | 0.0022^##^ | 0.0030^##^ | 2.21 | 0.0043^**^ | 0.0117^*^ | | 0.68 |
| PE 16:1-18:1 | 740.5219 | 11.71 | [M-H]- | 0.0009 | 0.0022^##^ | 0.0030^##^ | 2.68 | 0.0152^*^ | 0.0298^*^ | | 0.67 |
| PE 16:1-18:2 | 740.5266 | 11.55 | [M-H]- | 0.0013 | 0.0022^##^ | 0.0030^##^ | 2.79 | 0.0022^**^ | 0.0086^**^ | | 0.56 |
| PE 16:1-20:4 | 726.5455 | 13.21 | [M-H]- | 0.0005 | 0.0022^##^ | 0.0030^##^ | 2.12 | 0.0022^**^ | 0.0086^**^ | | 0.68 |
| PE 16:1-22:6 | 726.5465 | 13.06 | [M-H]- | 0.0011 | 0.0022^##^ | 0.0030^##^ | 2.58 | 0.0152^*^ | 0.0298^*^ | | 0.73 |
| PE 18:0-18:1 | 752.5591 | 12.85 | [M-H]- | 0.0021 | 0.0022^##^ | 0.0030^##^ | 2.26 | 0.0260^*^ | 0.0438^*^ | | 0.66 |
| PE 18:0-22:6 | 750.546 | 12.99 | [M-H]- | 0.0008 | 0.0022^##^ | 0.0030^##^ | 2.02 | 0.0043^**^ | 0.0117^*^ | | 0.78 |
| PE 18:1-18:1 | 748.5272 | 11.75 | [M-H]- | 0.0005 | 0.0022^##^ | 0.0030^##^ | 2.22 | 0.0022^**^ | 0.0086^**^ | | 0.71 |
| PE 18:1-18:2 | 748.5255 | 12.23 | [M-H]- | 0.0005 | 0.0022^##^ | 0.0030^##^ | 2.12 | 0.0022^**^ | 0.0086^**^ | | 0.70 |
| PE 18:1-20:4 | 760.4903 | 10.03 | [M-H]- | 0.0006 | 0.0022^##^ | 0.0030^##^ | 2.22 | 0.0022^**^ | 0.0086^**^ | | 0.64 |
| PE 18:1-20:5 | 746.5095 | 11.42 | [M-H]- | 0.0009 | 0.0022^##^ | 0.0030^##^ | 2.84 | 0.0043^**^ | 0.0117^*^ | | 0.59 |
| PE 14:1e/22:6 | 690.5106 | 12.15 | [M-H]- | 0.0014 | 0.0022^##^ | 0.0030^##^ | 2.27 | 0.0087^**^ | 0.0198^*^ | | 0.71 |
| PE 16:0e/20:4 | 744.5567 | 13.56 | [M-H]- | 0.0017 | 0.0022^##^ | 0.0030^##^ | 2.32 | 0.0087^**^ | 0.0198^*^ | | 0.67 |
| PE 16:0e/22:4 | 742.5378 | 12.46 | [M-H]- | 0.0008 | 0.0022^##^ | 0.0030^##^ | 2.30 | 0.0022^**^ | 0.0086^**^ | | 0.62 |
| PE 16:0e/22:6 | 728.5571 | 14.18 | [M-H]- | 0.0006 | 0.0022^##^ | 0.0030^##^ | 2.23 | 0.0022^**^ | 0.0086^**^ | | 0.67 |
| PE 16:1e/16:0 | 738.5051 | 11.23 | [M-H]- | 0.0009 | 0.0022^##^ | 0.0030^##^ | 2.28 | 0.0087^**^ | 0.0198^*^ | | 0.65 |
| PE 16:1e/16:1 | 724.5272 | 12.02 | [M-H]- | 0.0036 | 0.0022^##^ | 0.0030^##^ | 10.94 | 0.0260^*^ | 0.0438^*^ | | 0.58 |
| PE 16:1e/18:2 | 736.4918 | 10.49 | [M-H]- | 0.0005 | 0.0022^##^ | 0.0030^##^ | 2.36 | 0.0022^**^ | 0.0086^**^ | | 0.62 |
| PE 16:1e/20:1 | 736.4919 | 10.28 | [M-H]- | 0.0006 | 0.0022^##^ | 0.0030^##^ | 2.49 | 0.0022^**^ | 0.0086^**^ | | 0.61 |
| PE 16:1e/20:4 | 722.5146 | 11.75 | [M-H]- | 0.0015 | 0.0022^##^ | 0.0030^##^ | 2.04 | 0.0022^**^ | 0.0086^**^ | | 0.67 |
| PE 16:1e/22:1 | 718.4795 | 10.31 | [M-H]- | 0.0025 | 0.0022^##^ | 0.0030^##^ | 2.30 | 0.0260^*^ | 0.0438^*^ | | 0.69 |
| PE 16:1e/22:4 | 756.5898 | 15.35 | [M-H]- | 0.0017 | 0.0022^##^ | 0.0030^##^ | 2.18 | 0.0087^**^ | 0.0198^*^ | | 0.68 |
| PE 16:1e/24:4 | 766.535 | 12.07 | [M-H]- | 0.0006 | 0.0022^##^ | 0.0030^##^ | 2.61 | 0.0022^**^ | 0.0086^**^ | | 0.68 |
| PE 16:2e/22:6 | 766.5347 | 12.43 | [M-H]- | 0.0006 | 0.0022^##^ | 0.0030^##^ | 2.95 | 0.0043^**^ | 0.0117^*^ | | 0.58 |
| PE 18:0e/20:4 | 762.5052 | 10.95 | [M-H]- | 0.0009 | 0.0022^##^ | 0.0030^##^ | 2.05 | 0.0022^**^ | 0.0086^**^ | | 0.64 |
| PE 18:0e/22:6 | 748.5259 | 11.56 | [M-H]- | 0.0006 | 0.0022^##^ | 0.0030^##^ | 2.80 | 0.0022^**^ | 0.0086^**^ | | 0.62 |
| PE 18:1e/16:0 | 746.5162 | 11.03 | [M-H]- | 0.0008 | 0.0022^##^ | 0.0030^##^ | 2.32 | 0.0087^**^ | 0.0198^*^ | | 0.61 |
| PE 18:1e/18:2 | 744.5021 | 10.46 | [M-H]- | 0.0006 | 0.0022^##^ | 0.0030^##^ | 2.17 | 0.0022^**^ | 0.0086^**^ | | 0.72 |
| PE 18:1e/20:3 | 794.5739 | 13.31 | [M-H]- | 0.0015 | 0.0022^##^ | 0.0030^##^ | 2.07 | 0.0022^**^ | 0.0086^**^ | | 0.66 |
| PE 18:1e/20:4 | 780.5947 | 14.12 | [M-H]- | 0.0011 | 0.0022^##^ | 0.0030^##^ | 2.00 | 0.0022^**^ | 0.0086^**^ | | 0.66 |
| PE 18:1e/20:5 | 792.5499 | 12.48 | [M-H]- | 0.0006 | 0.0022^##^ | 0.0030^##^ | 2.32 | 0.0022^**^ | 0.0086^**^ | | 0.70 |
| PE 18:1e/22:5 | 778.5713 | 13.89 | [M-H]- | 0.0021 | 0.0022^##^ | 0.0030^##^ | 2.08 | 0.0152^*^ | 0.0298^*^ | | 0.67 |
| PE 18:2e/18:1 | 776.5557 | 12.8 | [M-H]- | 0.0011 | 0.0022^##^ | 0.0030^##^ | 2.25 | 0.0022^**^ | 0.0086^**^ | | 0.65 |
| PE 18:2e/20:1 | 776.5549 | 13.04 | [M-H]- | 0.0019 | 0.0022^##^ | 0.0030^##^ | 2.82 | 0.0043^**^ | 0.0117^*^ | | 0.64 |
| PE 18:2e/20:5 | 788.523 | 11.08 | [M-H]- | 0.0008 | 0.0022^##^ | 0.0030^##^ | 2.74 | 0.0022^**^ | 0.0086^**^ | | 0.65 |
| PE 18:2e/22:5 | 774.5475 | 12.56 | [M-H]- | 0.0011 | 0.0022^##^ | 0.0030^##^ | 2.91 | 0.0022^**^ | 0.0086^**^ | | 0.53 |
| PE 18:2e/22:6 | 774.5474 | 11.96 | [M-H]- | 0.0017 | 0.0022^##^ | 0.0030^##^ | 3.02 | 0.0022^**^ | 0.0086^**^ | | 0.67 |
| PE 18:3e/22:6 | 770.5149 | 10.72 | [M-H]- | 0.0009 | 0.0022^##^ | 0.0030^##^ | 2.75 | 0.0043^**^ | 0.0117^*^ | | 0.55 |
| PG 16:0-20:5 | 775.5493 | 11.23 | [M-H]- | 0.0030 | 0.0022^##^ | 0.0030^##^ | 2.72 | 0.0260^*^ | 0.0438^*^ | | 0.60 |
| PI 16:0-18:2 | 833.5162 | 9.31 | [M-H]- | 0.0008 | 0.0022^##^ | 0.0030^##^ | 2.06 | 0.0043^**^ | 0.0117^*^ | | 0.66 |
| PI 16:0-20:4 | 859.5342 | 9.53 | [M-H]- | 0.0009 | 0.0022^##^ | 0.0030^##^ | 2.08 | 0.0022^**^ | 0.0086^**^ | | 0.68 |
| PI 16:0-22:5 | 871.5364 | 9.65 | [M-H]- | 0.0005 | 0.0022^##^ | 0.0030^##^ | 2.06 | 0.0022^**^ | 0.0086^**^ | | 0.64 |
| PI 18:0-20:3 | 885.5509 | 10.11 | [M-H]- | 0.0008 | 0.0022^##^ | 0.0030^##^ | 2.34 | 0.0043^**^ | 0.0117^*^ | | 0.68 |
| PI 18:0-20:5 | 883.5304 | 9.54 | [M-H]- | 0.0009 | 0.0022^##^ | 0.0030^##^ | 2.72 | 0.0152^*^ | 0.0298^*^ | | 0.68 |
| PI 18:0-22:5 | 911.566 | 10.17 | [M-H]- | 0.0006 | 0.0022^##^ | 0.0030^##^ | 2.25 | 0.0022^**^ | 0.0086^**^ | | 0.64 |
| PS 16:0-18:1 | 760.5124 | 10.23 | [M-H]- | 0.0027 | 0.0022^##^ | 0.0030^##^ | 3.01 | 0.0043^**^ | 0.0117^*^ | | 0.52 |
| PS 18:0-16:1 | 760.5123 | 10.19 | [M-H]- | 0.0005 | 0.0022^##^ | 0.0030^##^ | 2.70 | 0.0022^**^ | 0.0086^**^ | | 0.66 |
| PS 18:0-18:1 | 788.5452 | 11.14 | [M-H]- | 0.0021 | 0.0022^##^ | 0.0030^##^ | 2.03 | 0.0152^*^ | 0.0298^*^ | | 0.61 |
| PS 18:0-20:4 | 810.5297 | 10.25 | [M-H]- | 0.0009 | 0.0022^##^ | 0.0030^##^ | 2.05 | 0.0043^**^ | 0.0117^*^ | | 0.69 |
| Cer-NDS d18:0/16:0 | 538.5197 | 12.66 | [M-H]- | 0.0011 | 0.0022^##^ | 0.0030^##^ | 3.52 | 0.0152^*^ | 0.0298^*^ | | 0.56 |
| Cer-NS d18:1/22:0 | 564.5342 | 13.53 | [M-H]- | 0.0014 | 0.0022^##^ | 0.0030^##^ | 3.25 | 0.0152^*^ | 0.0298^*^ | | 0.62 |
| Cer-NS d18:2/16:0 | 646.6103 | 16.12 | [M-H]- | 0.0025 | 0.0022^##^ | 0.0030^##^ | 3.18 | 0.0260^*^ | 0.0438^*^ | | 0.54 |
| Cer-NS d18:2/24:1 | 644.5949 | 15.04 | [M-H]- | 0.0025 | 0.0022^##^ | 0.0030^##^ | 2.45 | 0.0260^*^ | 0.0438^*^ | | 0.61 |
| FA 20:3 | 305.2472 | 4.87 | [M-H]- | 0.0017 | 0.0022^##^ | 0.0030^##^ | 2.59 | 0.0260^*^ | 0.0438^*^ | | 0.61 |

Model/Control, ^#^ P<0.05; ^##^ P<0.01; HL Granule/Model, ^*^ P<0.05, ^**^ P<0.01; FDR: FDR-adjusted p value; The differential lipids are ranked by Fold Change.
